# Systematic Exploration of SARS-CoV-2 Adaptation to Vero E6, Vero E6/TMPRSS2, and Calu-3 Cells

**DOI:** 10.1093/gbe/evad035

**Published:** 2023-02-28

**Authors:** Pakorn Aiewsakun, Worakorn Phumiphanjarphak, Natali Ludowyke, Priyo Budi Purwono, Suwimon Manopwisedjaroen, Chanya Srisaowakarn, Supanuch Ekronarongchai, Ampa Suksatu, Jirundon Yuvaniyama, Arunee Thitithanyanont

**Affiliations:** Department of Microbiology, Faculty of Science, Mahidol University, Bangkok, Thailand; Pornchai Matangkasombut Center for Microbial Genomics, Department of Microbiology, Faculty of Science, Mahidol University, Bangkok, Thailand; Department of Microbiology, Faculty of Science, Mahidol University, Bangkok, Thailand; Pornchai Matangkasombut Center for Microbial Genomics, Department of Microbiology, Faculty of Science, Mahidol University, Bangkok, Thailand; Department of Microbiology, Faculty of Science, Mahidol University, Bangkok, Thailand; Department of Microbiology, Faculty of Science, Mahidol University, Bangkok, Thailand; Department of Microbiology, Faculty of Science, Mahidol University, Bangkok, Thailand; Department of Microbiology, Faculty of Science, Mahidol University, Bangkok, Thailand; Department of Microbiology, Faculty of Science, Mahidol University, Bangkok, Thailand; Department of Microbiology, Faculty of Science, Mahidol University, Bangkok, Thailand; Department of Biochemistry and Center for Excellence in Protein and Enzyme Technology, Faculty of Science, Mahidol University, Bangkok, Thailand; Department of Microbiology, Faculty of Science, Mahidol University, Bangkok, Thailand; Pornchai Matangkasombut Center for Microbial Genomics, Department of Microbiology, Faculty of Science, Mahidol University, Bangkok, Thailand

**Keywords:** COVID-19, SARS-CoV-2, experimental evolutionary study, Vero E6, Vero E6/TMPRSS2, Calu-3

## Abstract

Severe acute respiratory syndrome coronavirus 2 (SARS-CoV-2) continues to spread globally, and scientists around the world are currently studying the virus intensively in order to fight against the on-going pandemic of the virus. To do so, SARS-CoV-2 is typically grown in the lab to generate viral stocks for various kinds of experimental investigations. However, accumulating evidence suggests that such viruses often undergo cell culture adaptation. Here, we systematically explored cell culture adaptation of two SARS-CoV-2 variants, namely the B.1.36.16 variant and the AY.30 variant, a sub lineage of the B.1.617.2 (Delta) variant, propagated in three different cell lines, including Vero E6, Vero E6/TMPRSS2, and Calu-3 cells. Our analyses detected numerous potential cell culture adaptation changes scattering across the entire virus genome, many of which could be found in naturally circulating isolates. Notable ones included mutations around the spike glycoprotein's multibasic cleavage site, and the Omicron-defining H655Y mutation on the spike glycoprotein, as well as mutations in the nucleocapsid protein's linker region, all of which were found to be Vero E6-specific. Our analyses also identified deletion mutations on the non-structural protein 1 and membrane glycoprotein as potential Calu-3-specific adaptation changes. S848C mutation on the non-structural protein 3, located to the protein's papain-like protease domain, was also identified as a potential adaptation change, found in viruses propagated in all three cell lines. Our results highlight SARS-CoV-2 high adaptability, emphasize the need to deep-sequence cultured viral samples when used in intricate and sensitive biological experiments, and illustrate the power of experimental evolutionary study in shedding lights on the virus evolutionary landscape.

**Significance**

Studies have shown that passaging severe acute respiratory syndrome coronavirus 2 (SARS-CoV-2) in cells lacking TMPRSS2 can induce adaptation on the virus spike glycoprotein's multibasic cleavage site (MBCS). However, systematic surveys of SARS-CoV-2 cell culture adaptation beyond this region are lacking. This study systematically investigated SARS-CoV-2 adaptation to Vero E6, Vero E6/TMPRSS2, and Calu-3 cells, and showed that different cell lines generated cell-line specific and potentially isolate specific adaptation mutations. The results confirmed the well-established cell-line adaptation mutations around the virus spike's MBCS, and also expanded the list to other sites that have been studied much less, or have not been reported before. This study highlights the virus high adaptability and emphasizes the need to deep-sequence cultured viral samples when used in sensitive biological experiments.

## Introduction

Severe acute respiratory syndrome coronavirus 2 (SARS-CoV-2) is a positive-sense single-stranded RNA virus that belongs to the *Coronaviridae* family in the order *Nidovirales*, genus *Betacoronavirus* (Gorbalenya et al. 2020). The virus is the causative agent of the coronavirus disease 2019 (COVID-19) that rapidly took over the globe, causing a worldwide pandemic (World Health Organization 2020) within just a couple of months after the initial report in December 2019 from China (Zhu et al. 2020).

RNA viruses are known for their high mutation rates, typically being in the order of 10^−4^ mutations per site per replication cycle (Sanjuán 2012). These are about four orders of magnitude greater than those of cellular organisms, like human (estimated to be ∼3.0 × 10^−8^ mutations per site per generation; Xue et al. 2009). This low replication fidelity has been primarily attributed to the lack of the proofreading activity of their polymerases (Steinhauer et al. 1992), and this has been proposed to set an “error threshold” for the RNA virus genome size beyond which deleterious mutations may accumulate so much that they become lethal to the virus population as a whole, known as the “error catastrophe” hypothesis, explaining their relatively small genome sizes (<15 kb; Holmes 2003). SARS-CoV-2, and other coronaviruses, are unique among RNA viruses in that, unlike other RNA viruses, they possess an error correction ability during replication, mediated by 3′-to-5′ exonuclease proofreading enzymes (Robson et al. 2020; Domingo et al. 2021), and this makes their mutation rates much lower than those of other RNA viruses. For example, the rate of mutation of SARS-CoV-2, the subject of this study, has been estimated to be ∼1.3 × 10^−6^ mutations per site per infection cycle (Amicone et al. 2022), approximately two orders of magnitude lower than the typical RNA virus mutation rate. Such a low mutation rate is thought to allow coronaviruses to expand and maintain their genomes, and indeed, viruses in this group have one of the largest RNA genomes known to date, being about 30 kb long (Gorbalenya et al. 2006).

In any case, this low rate of mutation of SARS-CoV-2 does not translate to a low rate of evolution—the rate at which the viral population as a whole changes over time across multiple successive generations. In fact, the rate of evolution of SARS-CoV-2 is very much comparable to those of other RNA viruses, estimated to be 30.92 substitutions per genome per year (Hadfield et al. 2018) or about 1.03 × 10^−3^ substitutions per site per year. This relatively high evolutionary rate could be largely explained by two biological properties applied generally to a broad range of viruses—that a virus typically has a short generation time and that the number of virion particles produced in a single round of replication is typically large. Although an absolute mutation rate of a virus may be low, there will nearly always be some mutants existing in the virus population due to a large number of virions produced in a short period of time. This would then allow natural selection to operate efficiently, and in turn would allow the virus to rapidly explore its evolutionary landscape still. It has also been demonstrated in culture that coronaviruses, including SARS-CoV-2, can generate extensive and diverse recombination products during replication (Gribble et al. 2021), and this feature may facilitate natural selection even further. Indeed, within the period of just a few years of the pandemic, SARS-CoV-2 had been observed to diversify into thousands of distinct lineages (O’Toole et al. 2021), testifying its high evolving ability despite its relatively low mutation rate. As the pandemic continues to unfold, scientists around the globe are now surveying and researching the virus phenotypic and genotypic changes intensively in order to fight against the evolving virus.

An experimental study of a virus typically begins with propagating viruses in the lab to generate highly concentrated viral stocks for downstream experiments. Similar to its close relative SARS-CoV that caused the global outbreaks in 2002–2003, an early study rapidly identified that SARS-CoV-2 uses angiotensin converting enzyme II (ACE2) as the main cell entry receptor (Zhou et al. 2020). The protein is known to be highly expressed on the surface of African green monkey's renal epithelium Vero E6 cells (Ren et al. 2006), and hence, the cells are often used to propagate SARS-CoV-2 in the lab. This practice, however, has been reported to rapidly induce mutations on the virus spike glycoprotein, specifically around its multibasic cleavage site (MBCS) _682_RxxR_685_ motif (Davidson et al. 2020; Klimstra et al. 2020; Lau et al. 2020; Liu et al. 2020; Ogando et al. 2020; Funnell et al. 2021; Lamers et al. 2021; Chaudhry et al. 2022). Spike glycoprotein is the primary viral protein that determines cell specificity and entry, directly binding to the ACE2 host cell receptor, and the MBCS is a unique feature of SARS-CoV-2's spike glycoprotein that those of its close relatives like SARS-CoV lack (Andersen et al. 2020), known to influence the mode of virus cell entry (see below; Mykytyn et al. 2021). Given its uniqueness and crucial roles in virus biology, it is thus possible that SARS-CoV-2 cultured in Vero E6 cells, which tends to harbor MBCS mutations in their spike glycoprotein, might not have authentic characteristics of the natural counterparts, and this potentially renders them unsuitable for various kinds of intricate and sensitive biological experiments.

It has been suggested that the MBCS mutations observed in culture are correlated with the availability of the virus cell entry pathway on the host target cells (Lamers et al. 2021). Two major distinct pathways of cell entry could be employed by SARS-CoV-2, including the serine protease-mediated pathway and the cathepsin-mediated pathway (reviewed in Jackson et al. 2022). In the former, after the engagement between the virus spike glycoprotein and ACE2, the spike glycoprotein is then cleaved by a host serine protease, such as transmembrane serine protease 2 (TMPRSS2), at the cell surface. This “activation” process exposes the spike glycoprotein's fusion peptide to the environment, which in turn enables membrane fusion to occur, allowing the virus to release its genome into the host cell. In the latter pathway, the virus is first internalized by endocytosis, and the spike glycoprotein is then activated by acid-activated cathepsins within the endolysosomes from which the virus releases its genome into the cell. It has been shown that when the host target cells lack or have insufficient TMPRSS2, the virus utilizes the endosomal cathepsin-mediated pathway to enter the cell, whereas when both proteases are available, the virus enters the cells using the cell-surface serine protease-mediated pathway, which is faster (Koch et al. 2021). MBCS has been shown to facilitate serine protease-mediated cell entry and decrease cathepsin-mediated protein activation (Mykytyn et al. 2021). In fact, the motif has been shown to even impair SARS-CoV-2 replication in Vero E6 cells, in which the cell-surface serine protease-mediated entry pathway is not available, while provides selective advantage in lung and primary human airway epithelial cells, which express TMPRSS2 (Peacock et al. 2021). These results supported that the MBCS motif is likely an adaptation to the cell-surface serine protease-mediated cell entry mode. Furthermore, it has been demonstrated that propagating Vero E6-propagated SARS-CoV-2 in human airway systems, such as in human pulmonary epithelium Calu-3 cells and human airway organoids, which naturally express serine proteases, could reduce the frequency of the MBCS mutations (Lamers et al. 2021). This again testified the rapid evolving ability of the virus, and further corroborated that the presence of TMPRSS2 decreases the fitness of MBCS mutants.

Nevertheless, it has been noted that growing SARS-CoV-2 in Calu-3 produces lower yields of infectious viral particles than when using Vero E6 (Matsuyama et al. 2020; de Souza et al. 2021). An alternative cell line is Vero E6/TMPRSS2, which is the standard Vero E6 cell line but with TMPRSS2 ectopically expressed on the cell surface. A study showed that infectivity of SARS-CoV-2 on Vero E6/TMPRSS2 cells is higher than those on Vero E6 and Calu-3 cells (Matsuyama et al. 2020). Furthermore, similar to Calu-3, propagating SARS-CoV-2 obtained from a Vero E6 culture in Vero E6/TMPRSS2 cells has been shown to reduce the frequency of MBCS mutations as a whole (Lamers et al. 2021). Thus, these cells might be preferred over Vero E6 cells for SARS-CoV-2 propagation to avoid cell culture adaptation at the MBCS motif.

Although SARS-CoV-2 cell culture adaptation at the spike glycoprotein's MBCS has been well documented, information on the virus cell culture adaptation beyond this region is lacking. A previous study detected mutations outside the spike glycoprotein coding region in cultured samples of SARS-CoV (Eckerle et al. 2010). An expansion of Vero E6-propagated SARS-CoV-2 in human airway organoids has also been noted to introduce new (minor) mutations to the virus that were not in the spike glycoprotein coding region (Lamers et al. 2021). All these results hinted at a possibility that coronavirus cell culture adaptation could occur elsewhere as well, and it is therefore important to systematically explore mutations across the entire genome and not just those in the spike glycoprotein coding region. Moreover, previous studies on this matter mostly used only viral samples collected in the early phase of the pandemic in 2020 (Davidson et al. 2020; Klimstra et al. 2020; Lau et al. 2020; Liu et al. 2020; Ogando et al. 2020; Lamers et al. 2021; Chaudhry et al. 2022). Thus, information on how different virus variants adapt to cell culture differently (or not) is still lacking.

In this study, we systematically explored cell culture adaptation of two SARS-CoV-2 variants across their entire genome, namely the Delta variant, which dominated the world in 2021, and the B.1.36.16 variant, which is genetically relatively more similar to the virus detected in the initial outbreaks in China. Adaptation of the viruses was examined in three different cell lines often used to grow SARS-CoV-2 in the lab, including Vero E6, Vero E6/TMPRSS2, and Calu-3 cells. Our analyses revealed that both SARS-CoV-2 variants are highly adaptive. Cell culture adaptation does occur beyond the spike protein's MBCS, and adaptive changes appeared to be variant- and cell-line specific. Overall, compared to Vero E6 and Calu-3 cells, we found that Vero E6/TMPRSS2 cells induced minimal culture adaptation for both virus variants, and thus the cell might be the best option among the three cell lines for SARS-CoV-2 culture and experimental investigations.

## Results and Discussion

### SARS-CoV-2 Serial Cell Culture Propagation Experiments

Four SARS-CoV-2 samples were propagated in Vero E6, Vero E6/TMPRSS2, and Calu-3 cells. Two were of the B.1.36.16 variant, namely CV130 (collection date: January 2021) and 73NLt (March 2021), and the other two were of the Delta AY.30 variant, namely NH783 (June 2021) and OTV54 (September 2021). All samples were obtained from COVID-19 patients in Thailand. The host cells’ expression levels of ACE2 and TMPRSS2 were quantified using immunofluorescence assay. All cell lines were found to have comparable levels of ACE2 expression. Vero E6/TMPRSS2 and Calu-3 cells showed comparably high levels of TMPRSS2 expression, while Vero E6 cells showed a significantly lower level of TMPRSS2 expression as expected (supplementary fig. S1, Supplementary Material online). These results corroborated well with previous published data (Ren et al. 2006; Aguiar et al. 2020; Matsuyama et al. 2020; Koch et al. 2021; Criscuolo et al. 2022; Zeng et al. 2022).

**Fig. 1.**
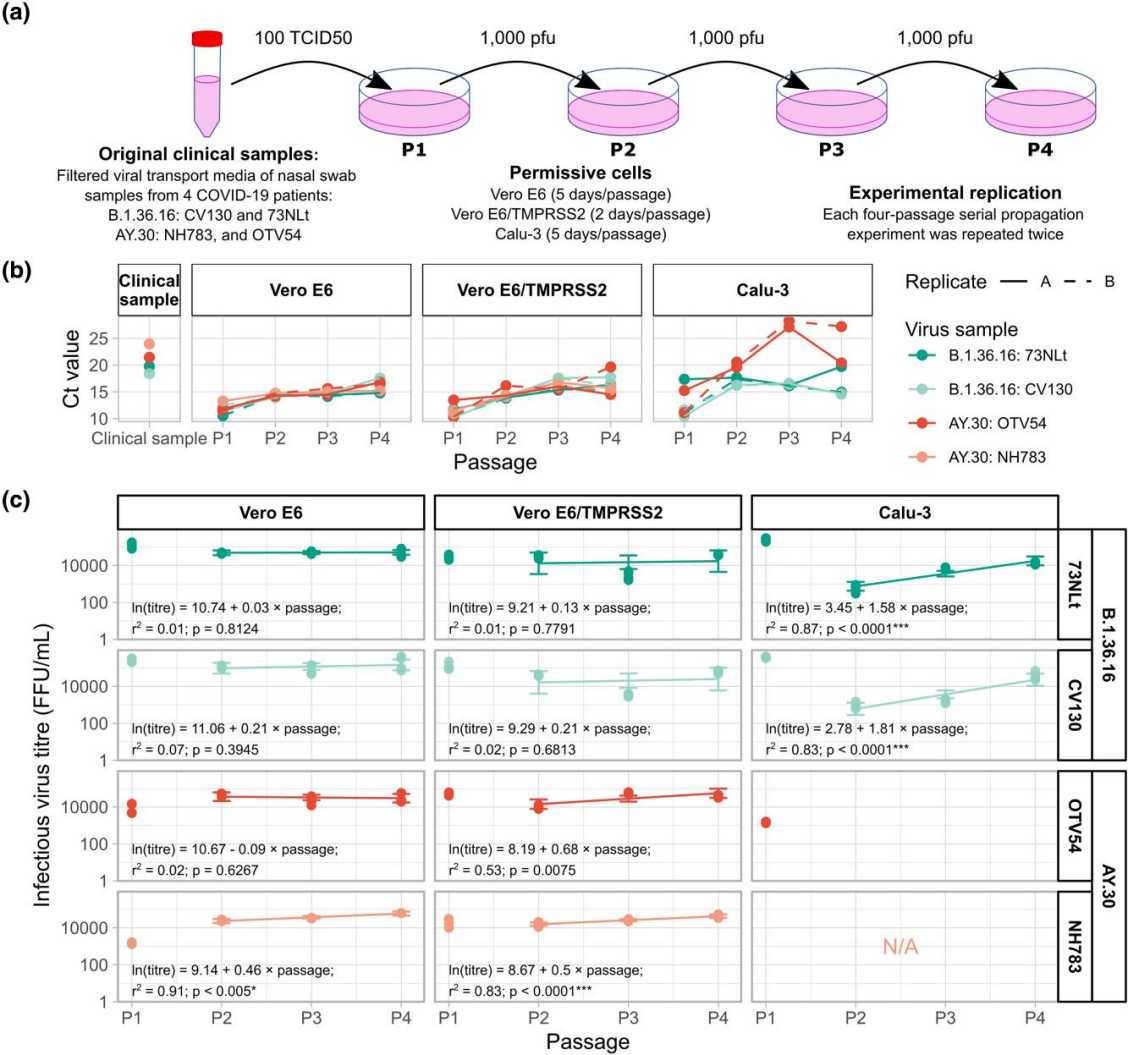
SARS-CoV-2 serial propagation experiments, qRT-PCR cycle threshold (*C_t_*) values, and infectious virus titers of the four viral samples propagated in Vero E6, Vero E6/TMPRSS2, and Calu-3 cells. (*a*) Two B.1.36.16 SARS-CoV-2 samples, CV130 and 73NLt, and two Delta AY.30 samples, NH783 and OTV54, were propagated in Vero E6, Vero E6/TMPRSS2, and Calu-3 cells for four passages, and each propagation experiment was performed in duplicate. (*b*) *C_t_* values of the four viral samples under the study (see key) in the original clinical samples (first panel from the left) and in the four passage stocks (passage 1–4: P1–4) propagated in the three different cell lines are shown. Raw *C_t_* values can be found in supplementary table S1, Supplementary Material online. (*c*) Infectious virus titers of P1–P4 of the four viral samples were evaluated using focus forming assay in Vero E6/TMPRSS2 cells, reported in the units of focus forming units per milliliter (FFU/ml). Each titration measurement was repeated twice using cell culture supernatants harvested from infected Vero E6, Vero E6/TMPRSS2, and Calu-3 cells at day 5, day 2, and day 5 post-infection, respectively (supplementary data S1, Supplementary Material online). Lines are linear models fitted to the log-transformed infectious virus titer data against time from P2 to P4 (time = 2, 3, and 4) aggregated across all replicates, using the *lm* function in R, reported with *P* values of the slope estimate, evaluated using the standard *t*-tests. Error bars show 95% confidence intervals of the estimated linear models at each timepoints. Bonferroni multiple testing corrected *P* value threshold of 0.05/10 tests = 0.005 was used to assess the slopes’ statistical significance; *P < 0.005; **P < 0.001; ***P < 0.0001.

Each propagation experiment was carried out for four passages and performed in duplicate (fig. 1*a*). In passage 1 (P1), the cells were inoculated with 100 units of 50% tissue culture infectious dose (TCID_50_) of SARS-CoV-2 containing virus transport media. For P2 to P4, the cells were inoculated with 1,000 plaque-forming units (PFUs) of the virus-containing supernatant from the previous passage, corresponding roughly to 0.001–0.00125 multiplicity of infection. For each passage, the viruses were grown for 5 days, 2 days, and 5 days in Vero E6, Vero E6/TMPRSS2, and Calu-3 cells, respectively, at which points clear cytopathic effects (CPEs) could be observed for all cultures and for both virus variants (i.e., grade 3+ or more; supplementary fig. S2, Supplementary Material online).

Quantitative reverse transcription polymerase chain reaction (qRT-PCR) were performed to quantify viral genomic materials in the clinical samples and in each passage stock (fig. 1*b* and supplementary table S1, Supplementary Material online). Infectious virus titers of each harvested passage viral stock were also quantified using focus forming assay in Vero E6/TMPRSS2 cells, with each measurement repeated twice (fig. 1*c* and supplementary data S1, Supplementary Material online). To assess if there was a significant increase in the titer as the propagation experiment progressed, a linear model was fitted to the log-transformed infectious virus titers against time from P2 to P4. The model fittings were performed separately for each viral sample propagated in each cell line. P1 titers were excluded since its seeding virus concentration (100 TCID_50_) was different from those of other passages (1,000 PFUs).

A previous study reported that at 2 days post-inoculation, SARS-CoV-2 RNA copies in the Vero E6/TMPRSS2 cell culture supernatants were much greater than those from VeroE6 cells and Calu-3 cells, and that Vero E6/TMPRSS2 displayed a much greater number of SARS-CoV-2 infected cells than Vero E6 (about 10 folds; Matsuyama et al. 2020). These results supported that Vero E6/TMPRSS2 cells are more susceptible to SARS-CoV-2 infection than the other two, in-line with our observation that it took longer for SARS-CoV-2 cultures in Vero E6 and Calu-3 cells to display clear CPEs than in Vero E6/TMPRSS2 cells. The fact that it took considerably shorter times to show clear CPEs in Vero E6/TMPRSS2 cells than Vero E6 cells was consistent with that TMPRSS2 alone could greatly enhance the cell susceptibility to SARS-CoV-2, in-line with the previous finding (Matsuyama et al. 2020).

All viral samples were successfully propagated for four passages with two duplicates in all cell lines, except for the AY.30 NH783 sample. In Vero E6 cells, we were able to complete the four-passage propagation experiment of the virus only once despite several attempts. In Calu-3 cells, we were unable to detect any infectious viral particles from the harvested supernatants as assessed by the classical plaque assay and focus forming assay, again despite several attempts and even with doubling the volume of the input virus. qRT-PCR analysis also failed to detect any viral RNA in the harvested cell lysates and cell supernatants. Paralleling this, although the AY.30 OTV54 sample could be propagated successfully in duplicate in Calu-3 cells, they did not grow very well. Its P2 to P4 infectious virus titers could not be measured by our focus forming assay (fig. 1*c*), indicative of low productions of infectious viral particles. Indeed, qRT-PCR (fig. 1*b* and supplementary table S1, Supplementary Material online) also revealed that the total viral RNA copies in these passage stocks, which may already include defective/non-assembled RNA viral genomes and those of defective/non-infectious viral particles, were considerably lower (*C_t_* value range: 19.58–28.28) than those of the B.1.36.16 samples (all *C_t_* values < 20). It was unclear why this was the case, but given that both NH783 and OTV54 samples were of the AY.30 variant, this difficulty in virus propagation appeared be an AY.30 variant-specific problem.

For those that infectious virus titers could be measured, a significant increase was observed for both of the B.1.36.16 samples propagated in Calu-3 cells, but not in Vero E6 or Vero E6/TMPRSS2 cells. For the AY.30 OTV54 sample, likewise, a significant increase in infectious virus titer could not be detected for the samples passaged in Vero E6 and Vero E6/TMPRSS2 cells. For the AY.30 NH783 sample, unlike other viral samples, a significant increase in its infectious virus titer could be detected in those propagated in Vero E6 and Vero E6/TMPRSS2 cells.

### Whole Genome Sequencing

**Fig. 2.**
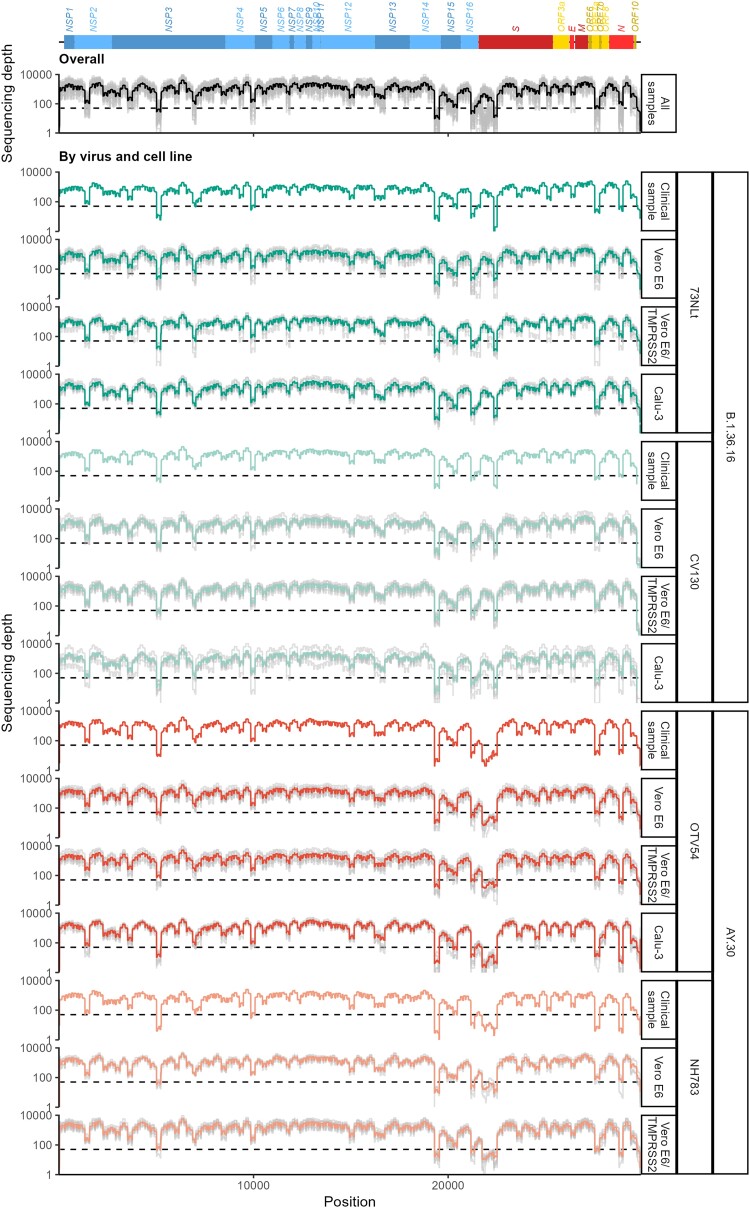
Site-wise sequencing depth. Sequencing depths (gray lines) were determined by mapping clean sequencing reads to the reference SARS-CoV-2 Wuhan-Hu-1 genome (RefSeq accession number: NC_045512.2). Site-wise sequencing depths averaged across all datasets (black), and those of each individual viral sample propagated in each cell line averaged across passage stocks, and experimental replicates are shown with colored lines. Horizontal dotted lines indicate a sequencing depth of 50×. SARS-CoV-2 genome structure is shown at the top, with coding region and gene names (blue: coding regions of NSPs; red: structural protein coding genes; yellow: accessory genes).

To investigate potential cell culture adaptation at the genomic level, original clinical samples and all passage stocks were deep-sequenced, using an amplicon-based sequencing approach with the ARTIC Network's V3 primer set (see Methods). The nucleic acid sequenced was obtained from 200 µl of culture supernatant combined with 50 µl of cell lysate. Sequencing reads were mapped to the Wuhan-Hu-1 SARS-CoV-2 reference genome (RefSeq accession number: NC_045512.2) to compute site-wise sequencing depth (and also site-wise variant profiles; supplementary data S2, Supplementary Material online). We found that the sequencing data covered most of the genome well (fig. 2), showing a grand average sequencing depth of 1,180.75× computed across all datasets (average depth by sample: 73NLt, 1,001.98×; CV130, 1,212.75×; OTV54, 1,216.19×; NH783, 1,394.86×). See supplementary table S1, Supplementary Material online for average depths, read numbers, and accession numbers of individual sequencing data.

Inspection of site-wise sequencing depths revealed several genomic regions with relatively low average sequencing depths (<50×), including the 5′ and 3′ untranslated regions (UTRs), some parts of the non-structural protein (NSP) 3, 14, 15, and 16 coding regions, as well as the N-terminal domain of the spike glycoprotein coding region, and some parts of the *ORF7a* and *ORF7b* genes (fig. 2, top). The patterns of “amplicon drop-off” were largely similar across all cell-line and viral sample datasets (fig. 2, bottom). The only notable difference was that while only the coding region of the 3′-end of the spike glycoprotein's N-terminal domain was poorly sequenced in B.1.36.16 samples, the entire coding region of the spike glycoprotein's N-terminal domain was poorly sequenced in both of the AY.30 samples, and this is known to be caused by the B.1.617.2 (Delta)-specific deletion at the positions 22,029–22,034 (Sanderson and Barrett 2021). This problem of amplicon drop-off is a known common issue of the sequencing approach we used, especially with the Delta variant (Itokawa et al. 2020; Lambisia et al. 2022). Analyses showed that this was largely due to dimer formations between amplification primers (Itokawa et al. 2020) and mutations in the primer binding sites (Kuchinski et al. 2022). It is also known that SARS-CoV-2 RNA genome has a complex secondary structure (Cao et al. 2021; Lan et al. 2022), and the virus can generate extensive and diverse recombination products with novel genetic combinations during replication in cell culture (Gribble et al. 2021). These biological features may also further obstruct binding of the amplification primers to the virus genomes, and thus might also contribute to the observed problem of amplification drop-off to some degree. Consequently, potential adaptive changes at these low sequencing depth loci could have been missed by this study. Nevertheless, our analysis still detected numerous potential cell culture adaptation mutations across the virus's entire genome (see below).

### Overall Intra-sample Genetic Diversity

Numbers of constant and polymorphic sites within each of the original and cultured stocks were counted (supplementary table S2, Supplementary Material online), excluding the first 30 bases of the 5′ UTR and the entire 3′ UTR, which were of poor sequencing depths (fig. 2). A site was considered a polymorphic site if it had two or more nucleotide variants with frequencies of >1% within the viral stock examined.

The numbers of polymorphic sites of the original clinical samples were estimated to be between 402 and 476 sites (median, 427.5 sites; standard deviation (SD), 37.3 sites), while those of cultured samples were between 161 and 1,163 sites (median, 402 sites; SD, 180.71 sites). This wider distribution of polymorphic site counts in cultured samples could be in part explained by the fact that in the propagation experiments, a relatively low number of viruses (1,000 PFUs of the virus-containing supernatant) was transferred over passages. This, in effect, stimulated founder effect/strong genetic drift phenomena, which are known to be associated with reduction in natural selection effectiveness and prolongment, if not accumulation, of deleterious mutations in the population (Livingstone 1969; Andrews 2010; Marsden et al. 2016). In addition, the sequenced genetic materials were obtained from culture supernatant and cell lysate fractions combined, which could potentially contain sequences of defective particles, non-infectious viral particles, and non-assembled viral RNA, likely contributing to the high number of polymorphic sites observed in cultured samples to some degree.

**Fig. 3.**
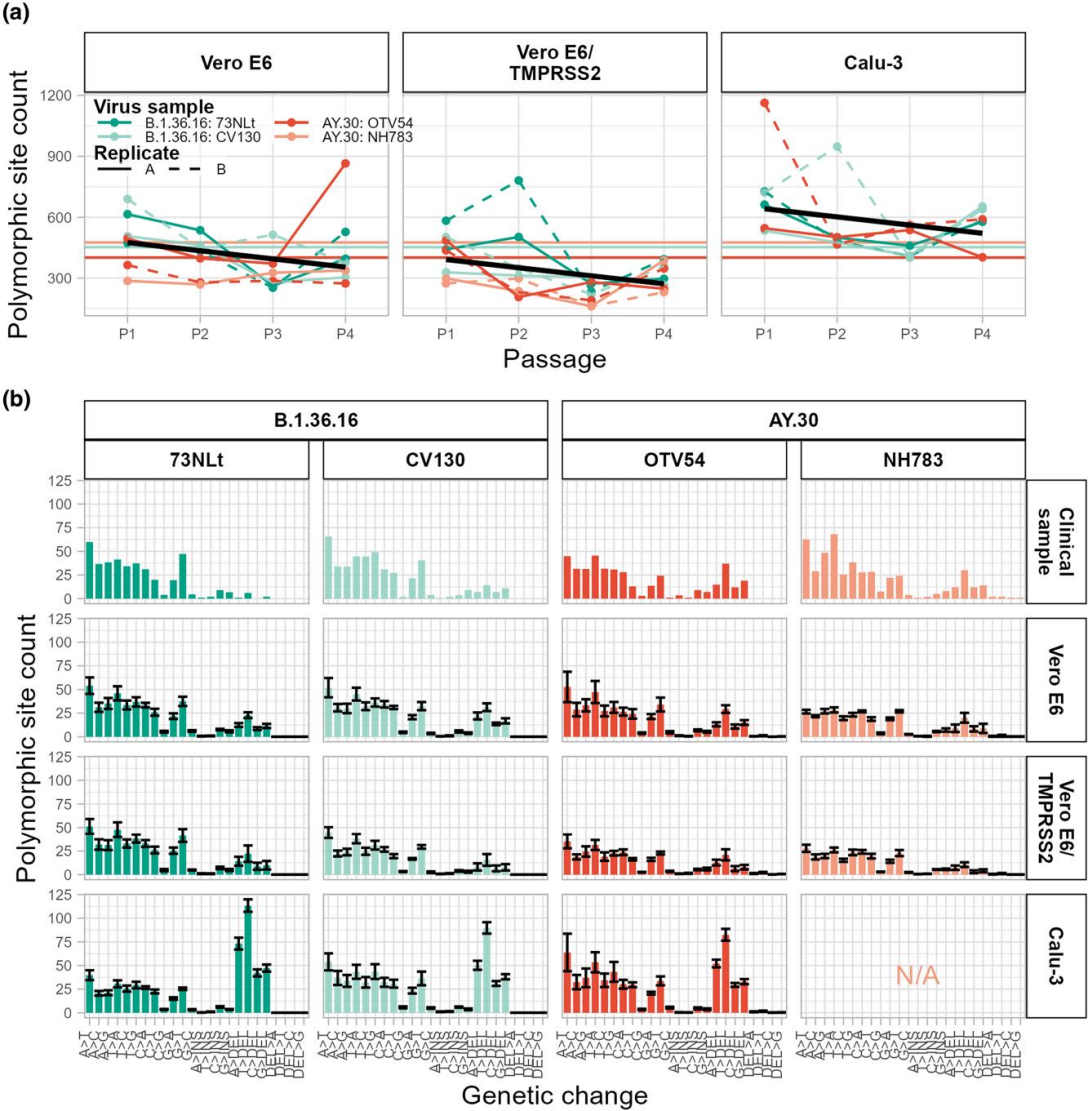
Polymorphic site counts through passages (*a*) and by genetic change type (*b*) in each virus sample propagated under various cell culture conditions. A site was considered polymorphic if it had two or more variants with frequencies of >1% within the examined viral stock, and the count excluded the first 30 bases of the 5′ UTR and the entire of 3′ UTR (229 sites) due to their low sequencing depths. (*a*) Polymorphic site counts through passages (supplementary table S2, Supplementary Material online). The dynamics are shown separately for the four viral samples and the two experimental replicates (see key). The number of polymorphic sites in the original clinical samples is indicated by horizontal dotted lines. Black solid lines are linear models describing the overall significant declining trends of the number of polymorphic sites through passages (count ∼ cell type + passage). Analysis suggested that the rates at which the number of sites decreased through passages were insignificantly different among viral samples and among cell lines used in the virus propagation, estimated to be about 40.22 sites per passage (standard error, 14.12 sites; *P* value, 0.00557). (*b*) Numbers of polymorphic sites by genetic change type (supplementary table S3, Supplementary Material online). The mean counts are shown together with standard errors. Six types of genetic variation were considered, including “A,” “T,” “C,” “G,” “DEL” (deletion), and “INS” (insertion). The direction of genetic change was inferred by assuming that the major variant detected in the original clinical sample was the original variant.

The numbers of polymorphic sites were highest in virus samples propagated in Calu-3 cells (range, 401–1,163 sites; median, 541.5 sites; SD, 176.88 sites), followed by those in Vero E6 cells (range, 252–865 sites; median, 386.0 sites; SD, 145.68 sites) and by Vero E6/TMPRSS2 cells (range, 161–781 sites, median, 298 sites; SD, 133.30 sites). Notably, the number of polymorphic sites appeared to significantly decrease through passages (fig. 3*a* and supplementary table S2, Supplementary Material online), approximately 40.22 sites per passage on average (standard error, 14.12 sites; *P* value, 0.00557) over the period of investigation. The rates at which the count decreased were neither significantly different among cell lines used in the propagation (linear regression with *F* test: H0 = count ∼ cell type + passage, H1 = count ∼ cell type * passage, *F* = 0.4234, df = 2, *P* = 0.6563) nor among viral samples (linear regression with *F* test: H0 = count ∼ cell type + passage, H1 = count ∼ cell type + passage + viral sample, *F* = 2.2217, df = 3, *P* = 0.0923). This general trend of declining polymorphic site counts through passages was consistent with selection at work. Most of the polymorphic sites found were bivariant sites (i.e., having only two variants at a minimum frequency of 1%), about 92.70% (range, 90.51–95.52%) in the original samples and about 96.04% (range, 88.05–99.38%) in cultured samples.

Assuming that the major variants found in the original clinical samples were the original variants, the types and directions of genetic changes at these polymorphic sites were inferred (fig. 3*b* and supplementary table S3, Supplementary Material online). We found that, overall, base substitutions and deletions were common, but insertions were rare. In fact, the high numbers of polymorphic sites observed in the samples grown in Calu-3 cells appeared to be substantially attributable to deletions, which are known to be associated with SARS-CoV-2 adaptation (McCarthy et al. 2021). Polymorphic site count distributions were found to be significantly different among viral samples and cell culture conditions (Poisson regression model fittings with likelihood ratio test: H0 = count ∼ variation type, H1 = count ∼ variation type + (viral sample + cell type), deviance = 2,320.9, df = 5, *P* < 0.0001), and the effects were not simply additive (H0 = count ∼ variation type + (viral sample + cell type), H1 = count ∼ variation type + (viral sample * cell type), deviance = 217.47, df = 5, *P* < 0.0001). The same results were observed even when polymorphic sites of the base substitution types were considered alone (fig. 3, 1st–9th bars; H0 = count ∼ variation type, H1 = count ∼ variation type + (viral sample + cell type), deviance = 536.44, df = 5, *P* < 0.0001; H0 = count ∼ variation type + (viral sample + cell type), H1 = count ∼ variation type + (viral sample * cell type), deviance = 469.98, df = 5, *P* < 0.0001). These results overall indicated differential mutation accumulations in different cell lines with a complex interaction between viral samples and cell lines. Proportionally, however, none of the base substitution count distributions were significantly different from each other (quasibinomial regression model fittings with likelihood ratio test: H0 = freq ∼ variation type, H1 = freq ∼ variation type + (viral sample + cell type), deviance = 8.8818 × 10^−16^, df = 5, *P* > 0.05), suggesting that although the absolute numbers of base substitutions may be different among viral samples grown in different cell lines, they were still generated by polymerases with the same intrinsic relative substitution error rates.

### Detection of Potential Cell Culture Adaptive Changes

For each cell-line dataset, at each individual site bearing mutations, we performed mixed-effects logistic regression analysis to estimate site-wise mutation selective advantage coefficients, Δs, for the two virus variants, while accounting for potential variations among viral samples, and experimental replicates, as well as genetic similarity among the viral samples. Δs is essentially a value that quantifies how much greater the intrinsic growth rate of the mutant virus is as compared to that of the original virus—a site with a positive Δs value is a site showing an overall increase in the frequency of the mutants over time (see Methods and supplementary notes, Material online for more details on mutation frequency temporal dynamic modeling as well as model fittings and comparisons). The major variant present in the original clinical sample was taken as the original variant, and any other variants were collectively grouped together as the mutant variant in the regression, as more than 96% of the polymorphic sites in cultured samples were bivariant (supplementary table S2, Supplementary Material online). At a site, we considered a virus variant to harbor potential adaptation mutations if its estimated Δs value was significantly greater than 0, and if any of its two viral samples had mutations at a collective frequency of >5% in any of the passage stocks in any of the experimental replicates. Such experiments were considered as showing strong signals supporting overall cell culture adaptation at that particular site regardless of the origins of the mutations (e.g., from infectious particles or otherwise).

**Fig. 4.**
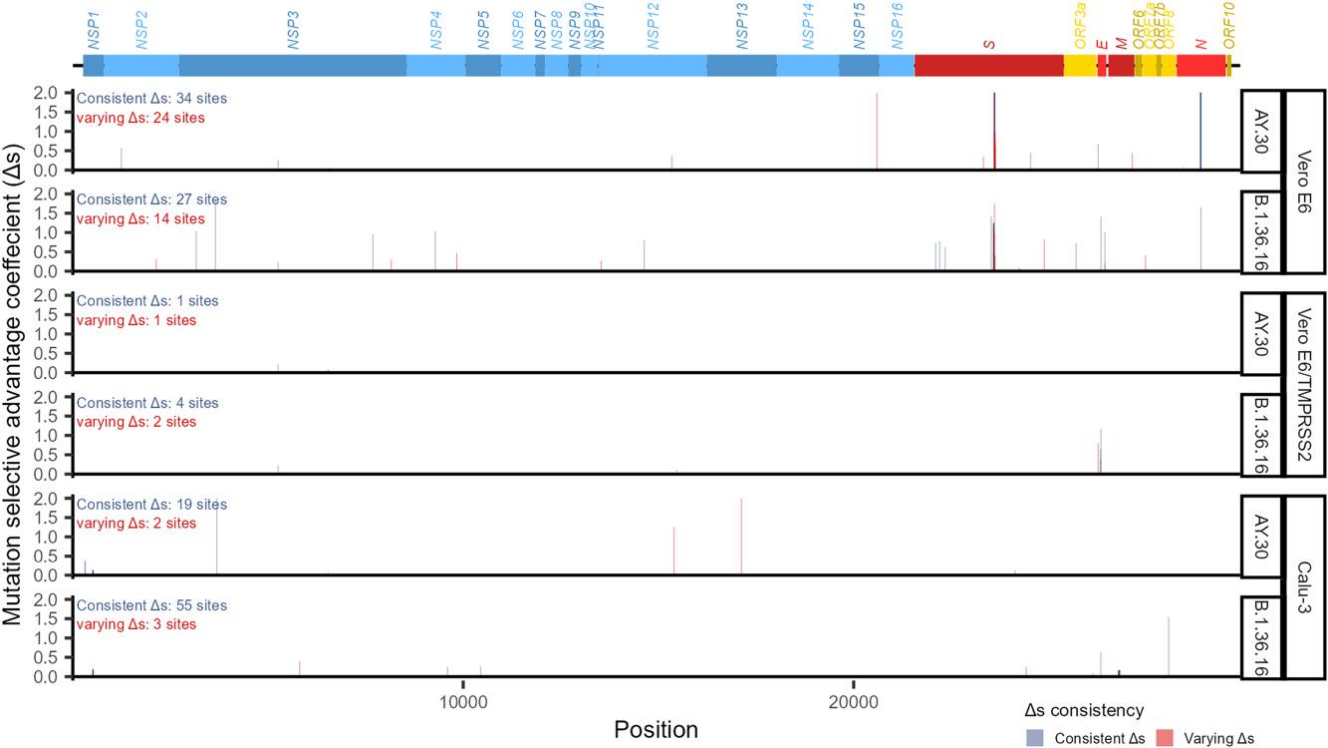
Sites detected as potentially harboring adaptation mutations. Site-wise mutation selective advantage coefficients, Δs, are shown, truncated at the maximum value of 2. The numbers of sites showing consistent Δs values (i.e., best described by the model *M1* in which Δs values are assumed to vary insignificantly among viral samples and experimental replicates, see Methods and supplementary notes, Supplementary Material online) are in blue, and those showing varying Δs values (i.e., best described by the model *M2* in which Δs values are allowed to vary significantly among viral samples and/or experimental replicates, see Methods and supplementary notes, Supplementary Material online) are in red. SARS-CoV-2 genome structure is shown at the top, with coding region and gene names (blue: coding regions of NSPs; red: structural protein coding genes; yellow: accessory genes).

Propagation of SARS-CoV-2 in all three cell lines appeared to be able to induce cell culture adaptation, although to a varying degree. Our analysis detected 157 positions scattering across the genome as bearing genetic variations with a significant Δs value for at least one virus variant in at least one propagation experiment (Vero E6, 90 sites; Vero E6/TMPRSS2, 7 sites; and Calu-3, 65 sites; fig. 4 and supplementary data S3, Supplementary Material online). Hundred and eighteen of the detected sites showed “consistent” Δs values, meaning that within the same virus variant, the Δs values were found to be insignificantly randomly varying among viral samples and experimental replicates, while 37 of them showed “varying” Δs values, meaning that the Δs values randomly vary significantly among viral samples and experimental replicates. Two sites showed both “consistent” and “varying” Δs values, depending on the cell types used in the virus propagation (site 6,539: Vero E6, varying Δs values; Vero E6/TMPRSS2, varying Δs values; and Calu-3, consistent Δs values; site 26,261: Vero E6, consistent Δs values; and Vero E6/TMPRSS2, varying Δs values). Supplementary figures S3–S5, Supplementary Material online shows mutation frequency dynamics at each of the detected sites. Some of these sites showed strong signals from multiple propagation experiments, and harbor changes of clear biological significance, including:

- a cluster of Vero E6-specific mutations at/around the spike protein's MBCS coding region,
- a Vero E6-specific non-synonymous change 23,525C > T in the *S* gene (H655Y),
- a number of Vero E6-specific mutations in the nucleocapsid protein's linker coding region (nucleotide (nt) positions 28,879–28,896, amino acid (aa) positions within the protein 203–208),
- a cluster of Calu-3-specific deletions in the NSP1 N-terminal domain coding region (nt positions 510–523, aa positions 82–86),
- a cluster of Calu-3-specific deletions in the coding region of the third transmembrane domain of the membrane glycoprotein (nt positions 26,787–26,816, aa position 86–98), and
- a non-synonymous change 5,262C > G in the NSP3 papain-like protease (PLpro) coding domain (S848C).

**Fig. 5.**
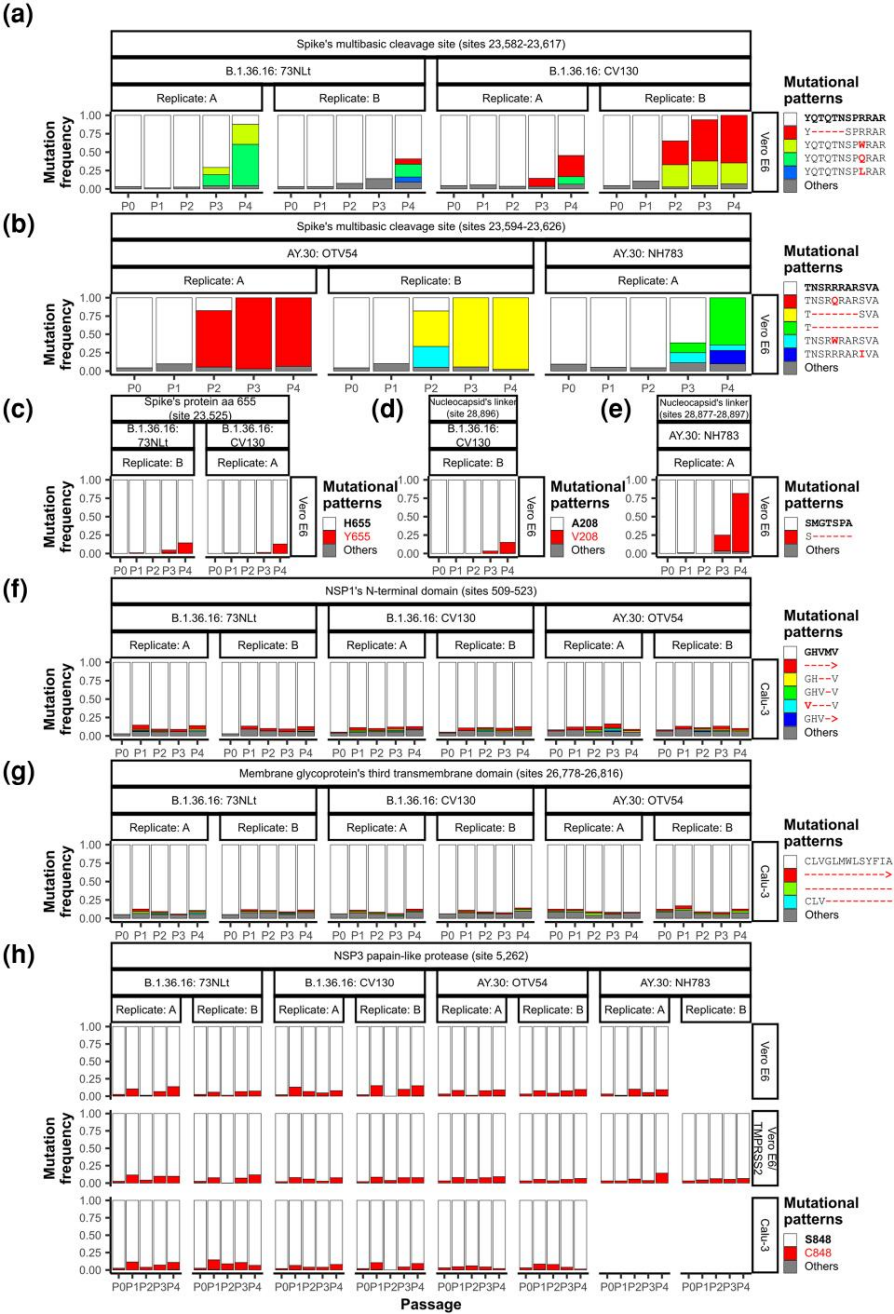
Temporal dynamics of the detected adaptive changes (*a* and *b*) around the spike protein's MBCS, (*c*) at the spike protein amino acid position 655, (*d* and *e*) in the nucleocapsid protein's linker domain, (*f*) in the NSP1 N-terminal domain, (*g*) in the 3^rd^ transmembrane domain of the membrane glycoprotein, and (*h*) in the NSP3 PLpro domain. First row of the strip labels above each plot indicates the locus at which potential adaption mutations were detected; second row indicates virus variant and the name of the virus sample; and third row indicates experimental replicate. The strip labels on the right-hand side of each plot indicate the cell line upon which the viruses were grown. Mutations around the spike protein's MBCS in B.1.36.16 (*a*) and AY.30 (*b*) were distinct, and thus are shown in separate plots. Likewise, the mutational profiles at the nucleocapsid protein's linker domain were distinct in B.1.36.16 (*d*) and AY.30 (*e*), and are shown in separate plots. In the figure legends, sequences found in the original clinical samples are in bold, and mutations with respect to the original sequences are highlighted in red. Arrow symbol (>) indicates a frameshift mutation. For deletion mutations, only reads spanning entire regions were used to compute the frequencies. See corresponding nucleotide mutational patterns in supplementary data, Supplementary Material online (*a* and *b*, supplementary data S4, Supplementary Material online; *e*, supplementary data S5, Supplementary Material online; *f*, supplementary data S6, Supplementary Material online; *g*, supplementary data S7, Supplementary Material online).

**Fig. 6.**
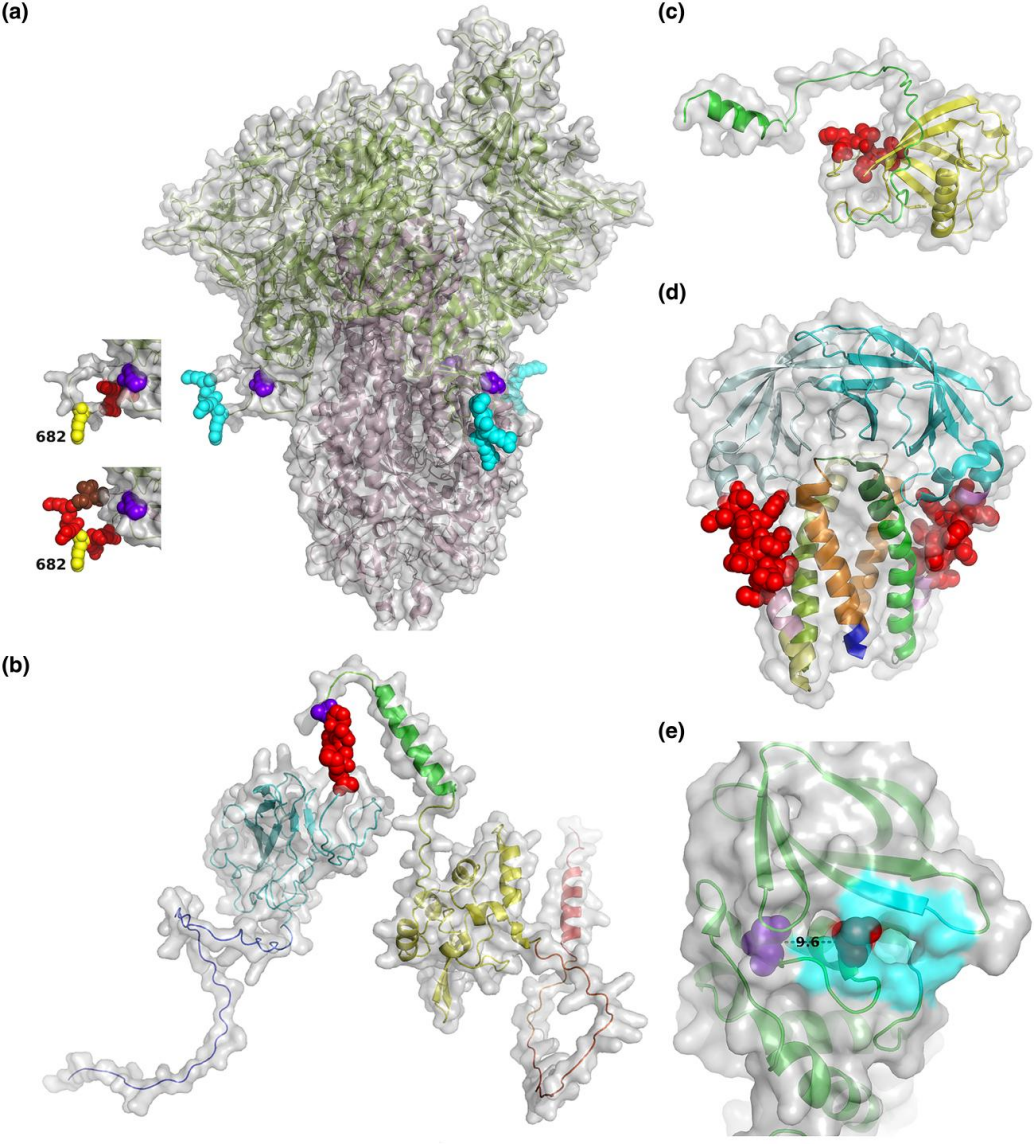
Positions of the focus adaptation mutations on the proteins: (*a*) spike glycoprotein, (*b*) nucleocapsid, (*c*) NSP1, (*d*) membrane glycoprotein, and (*e*) NSP3 PLpro. The AlphaFold2 (Jumper et al. 2021; Mirdita et al. 2022) models or Protein Data Bank structures 6vsb (Hwang et al. 2020), 8ctk (Dolan et al. 2022), or 7cjd (Gao et al. 2021) are drawn in cartoon representation with gray transparent surface. Positions harboring focused adaptation mutations (fig. 5) are shown as colored spheres using the PyMOL Molecular Graphics System, Version 2.5.4 (Schrödinger and DeLano 2020). (*a*) The RxxR motif (cyan) of the MBCS is located on a flexible loop, linking the S1 (pale green) and S2 (pale pink) portions of the trimeric spike. The top inset indicates the QTQTN sequence (red) and the R682 site (yellow), while the bottom inset shows the NSRxRAR (red), SVA (brown) sequence, and the R682 site (yellow). The H655 site is drawn in purple. (*b*) The SMGTSPA sequence in the linker domain of the nucleocapsid is depicted as red spheres with the A208 site colored purple. (*c*) The GHVMV sequence (red) is located on a flexible loop of the NSP1's N-terminal domain (yellow). The C-terminal sequence (green) forms a helix, found to be able to bind to the 40*S* ribosomal subunit and supposedly block its function (Schubert et al. 2020; Thoms et al. 2020). (*d*) The CLVGLMWLSYFIA sequence is shown as red spheres on the third transmembrane helices of the dimeric membrane glycoprotein. (*e*) The S848 site (purple) of NSP3 PLpro is 9.6 Å from the protease active site C855 (red), which is buried in the binding pocket (outlined with cyan surface).

Discussion focuses on these changes. See figure 5 for their temporal dynamics, and figure 6 for the position of the mutations within their associated proteins.

### Characterization of the Detected Potential Cell Culture Adaptive Changes

#### Mutations Around the Spike Protein's MBCS Coding Region

The two virus variants showed different mutational patterns around the spike protein's MBCS, but remarkably, samples of the same virus variant displayed similar patterns (supplementary data S4, Supplementary Material online and fig. 5*a* and *b*). For the B.1.36.16 samples, the main mutation found was a frame-preserving (although not in-frame) deletion of 15 nucleotides, 23,583_23,597delAT CAG ACT CAG ACT A, resulting in a deletion of five amino acids_675_QTQTN_679_ from the spike protein, Q675_N679del, just before the MBCS _682_RxxR_685_ motif (figs. 5*a* and 6*a*). The majority of the sites in this region were found to show consistent Δs values among viral samples and experimental replicates (supplementary data S3, Supplementary Material online). For the AY.30 samples, the main mutation found was a direct removal of the MBCS _682_RxxR_685_ motif itself (figs. 5*b* and 6*a*). The Δs values of the majority of the sites in this region, however, appeared to vary significantly among virus samples/experimental replicates (supplementary data S3, Supplementary Material online). R682W (23,606C > T) and R682Q|L (23,607G > A|T) were also detected as potential adaptation mutations in both of the virus variants propagated in Vero E6 cells (fig. 5*a* and *b*), although, again, their Δs values were found to vary significantly among viral samples/experimental replicates (supplementary data S3, Supplementary Material online). In any case, these mutations clearly persisted across multiple passages on the whole with frequencies generally increasing over time (Δs range: B.1.36.16, 0.38–1.75; AY.30, 0.28–3.57; supplementary data S3, Supplementary Material online), and often times driving the frequencies of the original variants down to <15%, and sometimes 0%, by P4. These results supported that these changes were genuine cell culture adaptation and are variant-specific, shaped by the virus prior genetic background. However, the fact that there were several adaptive mutational patterns detected at this location suggested that despite the said constraints, there likely are several convergent evolutionary pathways that the virus could take to adapt to Vero E6 cells here. This is also in-line with the well-established notion that an RNA viral population tend to be composed of “a cloud” of closely related but genetically distinct mutants due to their high mutability and evolving ability (Lauring and Andino 2010; Domingo and Perales 2019). These changes were not found in viruses propagated in Vero E6/TMPRSS2 and Calu-3 cells, supporting that these adaptive changes are Vero E6-specific, consistent with previous results (Davidson et al. 2020; Klimstra et al. 2020; Lau et al. 2020; Liu et al. 2020; Ogando et al. 2020; Funnell et al. 2021; Lamers et al. 2021; Chaudhry et al. 2022). In fact, it has been demonstrated that expansion of SARS-CoV-2 from Vero E6 stocks in Calu-3 cells, Vero E6/TMPRSS2 cells, and airway organoids could reduce the frequency of the MBCS mutants and increase the frequency of the virus bearing wild-type MBCS motif (Lamers et al. 2021), supporting that the MBCS mutations are actually even selected against in these cells.

The spike MBCS _682_RxxR_685_ motif, also known as the S1/S2 cleavage site, locates between the N-terminal receptor-binding S1 domain and the C-terminal fusion S2 domain of the spike protein (fig. 6*a*), and can be cleaved efficiently by host furin-like enzymes (Hoffmann, Kleine-Weber, Pöhlmann 2020; Peacock et al. 2021). This “priming” S1/S2 cleavage occurs during the spike protein biosynthesis within the host producer cell, exposing the S2 domain and another cleavage site called S2′ to the environment (Peacock et al. 2021). This would then allow another cut to happen at the S2′ site either by TMPRSS2 (Hoffmann, Kleine-Weber, Schroeder, et al. 2020) or by cathepsin L (Zhao et al. 2021) on or within the host target cell during viral entry. This second cleavage “activates’ the spike protein, liberating the fusion peptide within the S2 domain to the environment, and in turn enabling virus-host membrane fusion, and cell entry, to occur. The former protease mediates a “fast” cell-surface cell entry route, while the latter mediates a “slow” endosomal entry pathway (Jackson et al. 2022; Takeda 2022). A study showed that when the host target cells lack TMPRSS2, the virus utilizes the slow endosomal pathway to enter the cell, whereas when both proteases are available, the virus uses the cell-surface entry pathway (Koch et al. 2021). In keeping with previous published data (Lamers et al. 2021), our results supported that when the virus is forced to enter cells using the endocytic entry pathway, such as in Vero E6 cells, mutations at, or around, the virus spike protein MBCS _682_RxxR_685_ motif are positively selected.

It is still unclear why an efficient S1/S2 cleavage would be disadvantageous in such a scenario. One possibility is that highly efficient S1/S2 cleavage may result in overly premature shedding of the S1 domain, and this could make cell attachment comparatively less stable, and subsequently reduce the chance of viral particles being endocytosed (Peacock et al. 2021). Studies have shown that the MBCS of the Delta variant with the P681R mutation is more efficient than the wild-type P681 version (Lubinski et al. 2022; Saito et al. 2022). According to this hypothesis, it is therefore expected that the Delta MBCS motif would be more deleterious than the wild-type one under this circumstance, and indeed, we observed a complete deletion of the _682_RxxR_685_ motif in the Delta AY.30 samples, and not in the B.1.36.16 samples, which had the wild-type P681 MBCS variant. AY.30's site-wise Δs values were also estimated to be generally greater than that of B.1.36.16 at this region, further corroborating this hypothesis. This hypothesis could also explain the difficulty of propagating the Delta AY.30 NH783 sample in Vero E6 cells, and the low infectious virus titers of the AY.30 samples in P1 (fig. 1*c* and supplementary data S1, Supplementary Material online; range: 1,300–15,000 foci-forming unit (FFU)/ml) compared to those of the B.1.36.16 samples (range: 80,000–330,000 FFU/ml).

#### H655Y Mutation on the Spike Protein

This mutation was again found to be Vero E6-specific. Strong positive selection signals were detected in two B.1.36.16 propagation experiments, in which the P4 mutation frequencies were found to be between 13% and 14% (fig. 5*c*). An overall Δs value was estimated to be 1.42 for the B.1.36.16 variant and was found to be insignificantly randomly varying among virus samples and experimental replicates (supplementary data S3, Supplementary Material online).

Intriguingly, this mutation is one of the defining mutations of the Omicron variant, which prefers cathepsin-dependent endosome entry (Yamamoto et al. 2022). It is in fact precisely this mutation that determines the virus preferential usage of the late endosomal pathway and impairs the cell-surface entry pathway (Yamamoto et al. 2022). This suggested that S1/S2 cleavage efficiency is not the only factor that modulates virus cell entry, and echoes the notion of the plurality of evolutionary pathways that the virus could take to adapt to its environment.

Structural examination suggested that H655Y mutation likely increases the stability of the S1/S2 complex, and hence the attachment of the virus to the host target cell, which would in turn allow for a more efficient cell internalization of the virion particles by endocytosis, promoting the virus cathepsin-dependent endosome entry (Yamamoto et al. 2022). This is essentially the other side of the hypothesis proposed to explain why an efficient S1/S2 cleavage site would be deleterious for viruses propagated in Vero E6 cells mentioned above.

In any case, despite the fact that all virus propagation experiments using Vero E6 cells resulted in adaptive changes in either the virus spike glycoprotein's MBCS domain or the H655Y mutation (fig. 5*a*–*c*), all except only one (the AY.30 NH783 sample) showed insignificant increases in infectious virus titer as the experiments progressed (fig. 1*c*). These adaptive changes thus unlikely directly affect the overall net rate of the production of SARS-CoV-2 infectious viral progenies.

#### Mutations on the Nucleocapsid Protein's Linker Domain

Several mutations on the nucleocapsid protein's linker domain were detected as potential Vero E6-specific adaptation from multiple experiments (figs. 5*d*, *e*, and 6*b*). The strongest ones included A208V (nt mutation, 28,896C > T; virus variant, B.1.36.16; virus sample, CV130; number of experimental replicates showing strong signal, one; Δs value, 1.66; P4 mutation frequency, 15%; fig. 5*d*) and M203_A208del (nt mutation, 28,879_28,896delT ATG GGA ACT TCT CCT GC; virus variant, AY.30; virus sample, NH783; number of experimental replicates showing strong signal, one; Δs value range, 2.28–2.91; P4 mutation frequency, 79.59%; fig. 5*e*). Site-wise Δs values were estimated to be statistically consistent among experimental replicates and among viral samples of the same virus variant (supplementary data S3, Supplementary Material online).

A study of SARS-CoV-2 circulating in humans by Wu et al. (2021) showed that mutations in nucleocapsid linker domain are common, in particular R203K and G204R. These mutations can be found in the B.1.1.7 (Alpha), P.1, P.2 (Zeta), P.3 (Theta), and C.37 (Lambda) variants, and they had been shown to provide a competitive advantage in the hamster model and human airway tissues. The study also reported that the mutant's infectivity was higher than that of the wild-type variant measured in Vero E6 and Calu-3 cells, and the mutations enhanced disease severity in the hamster model. Other studies later showed that R203K and G204R mutations can also be found in the B.1.1.529 and BA (Omicron) variants as well (Hossain et al. 2022; Ippoliti et al. 2022; Jung et al. 2022). While it is unclear if the mutations observed herein have similar effects to the R203K|G204R mutations, this finding warrants further investigation of the effects of mutations on nucleocapsid protein's linker domain on the virus.

#### Deletions in the NSP1 N-Terminal Domain

Driven mainly by the discovery of MBCS-associated Vero E6 adaptation mutations, Calu-3 cells have been proposed as an alternative to Vero E6 cells for SARS-CoV-2 propagation since such mutations are selected against in Calu-3 cells (Baczenas et al. 2021; Lamers et al. 2021; Chaudhry et al. 2022). We, indeed, did not detect any adaptation mutations in the spike protein's MBCS in viral samples propagated in Calu-3 cells, but our experiments nevertheless did reveal that propagating SARS-CoV-2 in Calu-3 could still induce mutations.

In both of the B.1.36.16 and AY.30 variants, our analysis detected deletions of various sizes in the NSP1's _82_GHVMV_86_ motif as potential Calu-3-specific adaptation mutations (figs. 5*f* and 6*c*). All sites were estimated to have positive Δs values that were insignificantly randomly varying among viral samples and experimental replicates (supplementary data S3, Supplementary Material online). This locus is in fact a known mutation hotspot, found in SARS-CoV-2 circulating in humans (Lin et al. 2021). These mutations, however, appeared to have relatively low selective advantage in our experiments (Δs range: 0.09–0.21; supplementary data S3, Supplementary Material online), and the frequencies of the original variant remained above 85% in all passages in all propagation experiments (fig. 5*f*). This pattern is therefore overall more consistent with a balancing selection scenario, in which multiple variants are actively maintained in a population.

NSP1 is composed of two protein domains—the globular N-terminal domain, and the C-terminal domain comprising two α helices—linked together by a flexible linker region (Schubert et al. 2020; Thoms et al. 2020). The main function of NSP1 is to inhibit the host protein synthesis by inserting its C-terminus into the mRNA entry tunnel on the ribosome, and thereby “shutoff” the host protein translation process (Schubert et al. 2020; Thoms et al. 2020). The N-terminal domain, nevertheless, has also been demonstrated to contribute to this process to some extent (Mendez et al. 2021; Kumar et al. 2022). In particular, deletion mutations at this location have been shown to result in a lower level of mRNA translation inhibition (Kumar et al. 2022), and alter the host transcriptome profiles to be more like the negative control one, although still distinct (Lin et al. 2021), likely due to the protein structure destabilization (Lin et al. 2021; Kumar et al. 2022). Perhaps due to its potent negative effect on the host gene expression system, NSP1 has been shown to cause the most severe viability reduction in the human lung H1299 cells compared to other SARS-CoV-2 NSPs (Yuan et al. 2020). Cell viability reduction could also be observed in Vero E6 cells although to a lesser extent (Yuan et al. 2020). This might partly explain our observed difficulty in propagating SARS-CoV-2 in Calu-3 cells compared to Vero E6 cells, and also the previous observations of fewer infectious viral particles produced by Calu-3 cells (Matsuyama et al. 2020; de Souza et al. 2021).

#### Deletion in the Membrane Glycoprotein's Third Transmembrane Domain

Another detected Calu-3-specific adaptation mutation was a deletion at the genomic positions 26,778–26,816, locating to the third transmembrane domain of the membrane glycoprotein (figs. 5*g* and 6*d*; aa positions 86–98; Bianchi et al. 2020; Mahtarin et al. 2020; Thomas 2020). Significantly positive site-wise Δs values were detected only from the B.1.36.16 samples, and were found to be insignificantly randomly varying among virus samples and experimental replicates, but similar to the deletions in the NSP1's N-terminal domain, the absolute selection strength appeared to be relatively low (Δs range: 0.14–0.17; supplementary data S3, Supplementary Material online). Examination of the raw read mappings revealed that although the estimated site-wise Δs values were not significantly >0, the AY.30 OTV54 sample propagated in Calu-3 cells actually also harbored and maintained similar deletions at this locus. The frequencies of the original variant remained above 80% in all passages in all propagation experiments (fig. 5*g*), again, together suggestive of balancing selection.

Within the host producer cell, membrane proteins interact with many viral proteins, playing several important roles in virus budding and particle formation (Alsaadi and Jones 2019; Wong and Saier 2021). Boson et al. (2021) showed in Vero E6 cells that membrane protein can induce retention of the virus spike glycoprotein within the cell, and this prevents spike glycoproteins from accumulating on the cell surface, which in turn reduces the cell–cell fusion and syncytia formation. They also showed that the last 19 amino acids of the spike glycoprotein in its cytoplasmic tail are essential for the membrane protein-mediated spike glycoprotein retention. Coincidentally, many of the amino acid residues surrounding and within the deleted region detected in the membrane protein have been predicted as being involved in its interaction with the spike glycoprotein's C-terminal part (Mahtarin et al. 2020). It could thus be hypothesized that this deletion might interfere with the membrane protein-mediated retention of the spike glycoprotein, which then might affect the rate of cell–cell fusion and syncytia formation. This finding warrants further experiments to investigate if this was the case in Calu-3 cells or not, and if so, how it would affect the virus replication and life cycle in culture.

#### S848C Mutation on NSP3 PLpro

This mutation was detected as potential cell culture adaptation in both the B.1.36.16 and AY.30 variants propagated in Vero E6 and Vero E6/TMPRSS2 cells (figs. 5*h* and 6*e*). The estimated site-wise Δs values for the two virus variants were found to be insignificantly randomly varying among virus samples and experimental replicates, but the estimated values were relatively low (Δs range: 0.22–0.27; supplementary data S3, Supplementary Material online), and the mutation frequencies did not go above 15% in any of the propagation experiments, fluctuating at around 5–6%. In fact, viruses propagated in Calu-3 cells also displayed this mutation with similar frequencies (fig. 5*h*), but their estimated Δs values were not significantly >0. Combined, these results, again, were overall consistent with balancing selection.

PLpro is a highly conserved protein among coronaviruses. In SARS-CoV, the protein has been shown to play an essential role in processing viral polyproteins (Harcourt et al. 2004). In SARS-CoV-2, the protein has been shown to regulate the host antiviral innate immunity by contributing to the cleavage of the ubiquitin-like interferon-stimulated gene 15 protein from interferon responsive factor 3, and the attenuation of type I interferon responses (Shin et al. 2020). Due to its multi-vital functions, PLpro is an attractive pharmaceutical target, and numerous studies have been conducted to identify the protein's inhibitors (see Anirudhan et al. 2021; Calleja et al. 2022; Jiang et al. 2022 for reviews). The amino acid residue S848 locates closely to the protein catalytic pocket and active site C856 (or C111 of the NSP3 PLpro domain alone; Klemm et al. 2020; Rut et al. 2020; Gao et al. 2021), being just about 9.6 Å apart (fig. 6*e*). This warrants further studies to explore potential effects of the mutation on the protein and biological effects on the virus.

#### Other Potential Cell Culture Adaptation Mutations

Besides the mutations discussed above, numerous other mutations were also detected as potential cell culture adaptive changes by our analysis (fig. 4 and supplementary data S3, Supplementary Material online), including synonymous and non-synonymous changes in the coding regions of NSP2, other domains of NSP3 outside the PLpro domain, NSP4, NSP5, NSP12, NSP13, NSP15, other domains of spike glycoprotein beyond the MBCS domain, ORF3a protein, envelop protein, the C-terminal domain of the membrane glycoprotein, ORF7a protein, the RNA-binding motif in the N-terminal domain of the nucleocapsid protein, and ORF8 protein. Viruses propagated in the three different cell lines harbored different sets of these mutations, but all appeared to bear some. Majority of these changes, however, showed sporadic adaptation signals (i.e., a strong signal could be detected in only one virus propagation experiment; supplementary data S3, Supplementary Material online); thus, evidence of cell culture adaptation of these changes was inconclusive. Nevertheless, these observations revealed that the virus genome is highly plastic, highly adaptive, and shed light on the biological viable regions of SARS-CoV-2 evolutionary landscape.

## Conclusion and Final Remarks

This study demonstrated that SARS-CoV-2 is able to adapt to its environment extremely rapidly, paralleling the fact that, as the pandemic unfolds, new SARS-CoV-2 variants continue to emerge and to be discovered. Many of the cell culture adaptation mutations appeared to be maintained in the cultures by balancing selection, and were detected at the frequencies of <50%, meaning that they would not show in the consensus sequences. Deep sequencing should therefore be applied when cultured viruses are used in experimental investigation to make sure that the virus has the expected biological characteristics.

Overall, compared with Vero E6 and Calu-3 cells, we found that Vero E6/TMPRSS2 cells induced minimal culture adaptations, and thus the cell might be the best option among the three cell lines for SARS-CoV-2 culture and experimental investigations. Nevertheless, a number of potential cell culture adaptation mutations could still be detected in viruses propagated in Vero E6/TMPRSS2 cells; thus, caution should be exercised when using cultured viruses in highly intricate and sensitive biological experiments. It is unclear if these potentially cell culture adaptation mutations affect, for example, vaccine effectiveness and antiviral drug screening and testing or not, in which virology is of a highly important and relevant matter. The results from this study warrant further research to investigate this matter.

In addition, the recurring theme of the results we observed herein was that many of the mutations detected are also naturally occurring mutations. Despite the limited number of viral samples investigated, this study thus unequivocally illustrated that experimental evolutionary study is an extremely powerful tool for studying viruses, allowing us to get a glimpse into biological viable regions of their evolutionary landscape.

## Methods

### SARS-CoV-2 Viral Samples

Nasopharyngeal specimens were collected from patients at Chakri Naruebodindra Medical Institute, Faculty of Medicine Ramathibodi Hospital, Mahidol University, Samut Prakan, Thailand, confirmed to be SARS-CoV-2 positive by qRT-PCR of the virus *ORF1ab* and *N* gene fragments (Sansure Biotech Inc., PR China). The samples were preserved in viral transport medium (VTM; universal transport medium, COPAN Diagnostics Inc., USA). Initially, the samples were centrifuged for 1 min at 10,000 rpm, and passed through a sterile 0.45 μm pore size syringe filter. Virus titers of the virus-containing VTM were measured in quadruplicate in 96-well microtiter plates on Vero E6/TMPRSS2 cells in serial dilution to obtain TCID_50_ using the Reed and Münch endpoint method (Reed and Muench 1938). All the experiments with live SARS-CoV-2 virus were conducted at a certified biosafety level 3 facility at the Department of Microbiology, Faculty of Science, Mahidol University, Thailand.

### Genotyping

Nucleic acid was extracted from 200 µl of VTM using the GenTiTM32 Automatic Extraction System (Advanced Viral DNA/RNA Extraction Kit) according to the manufacturer's instructions, followed by Sanger sequencing of a SARS-CoV-2 spike protein coding region as previously described (Daniels et al. 2021). Vector NTI® Contig Express software V1.5.1 was used to generate viral sequences from raw sequencing data, and Pangolin v3.1.14 (O’Toole et al. 2021) was used to genotype the viruses. The genotypes were confirmed by using analysis of whole genome consensus sequences.

### Cell Lines

African green monkey (*Cercopithecus aethiops*) kidney epithelial Vero E6 cells (ATCC®CRL-1586™) were obtained from the American Type Culture Collection (ATCC, USA). These cells were maintained in Dulbecco's modified eagle medium (DMEM) high glucose (Gibco, USA) with 10% fetal bovine serum (FBS; Gibco, USA) and 100 μg/ml penicillin/streptomycin (PS; Invitrogen, USA). Vero E6 cells with a stably high expression of TMPRSS2 (Vero E6/TMPRSS2 cells) were obtained from Japanese Collection of Research Bioresources (JCRB) Cell Bank, Japan (JCRB number: JCRB1819). The cells were cultured in DMEM low glucose (Gibco, USA), supplemented with 10% FBS (Gibco, USA), G418 (1 mg/ml; Nacalai Tesque, Japan), and 100 μg/ml PS (Invitrogen, USA). Vero cells (ATCC®CCL-81™) were brought from the ATCC (USA) and grown in minimum essential medium (MEM; Gibco, USA) with 10% FBS and 1× non-essential amino acids (MEM-NEAA; Gibco, USA) supplementation. Human airway epithelial cells Calu-3 were obtained from ATCC (ATCC®HTB-55™). The cells were cultured in DMEM: nutrient mixture F-12 (DMEM/F-12; Gibco, USA) with 10% FBS (Thermo Scientific Fisher, USA), 100 μg/ml PS (Invitrogen, USA), and 1% GlutaMAX (Gibco, USA). All cultures were maintained in a humidified incubator at 37 °C and with 5% CO_2_.

### Quantification of ACE2 and TMPRSS2 Expression Levels in Vero E6, Vero E6/TMPRSS2, and Calu-3 Cells using Immunofluorescence assay

Each cell line was seeded into wells in 96-well plates one day before the assay. Cells were fixed with 4% paraformaldehyde for 1 h at room temperature and blocked by 2% (w/v) bovine serum albumin in phosphate-buffered saline (PBS) with 0.5% Tween (PBST) for 1 h at room temperature. The cells were then incubated with the primary antibody, binding either to the ACE2 [rabbit anti-ACE2 polyclonal antibody (1:500) (Abcam, USA)] or TMPRSS2 [mouse anti-TMPRSS2 monoclonal antibody (1:50) (Santa Cruz Biotechnology, USA)] proteins, at 37 °C for 1 h. After that, the cells were washed with PBST three times, and incubated with the secondary antibody; goat anti-rabbit Alexa 488 (1:500; Invitrogen, USA) or goat anti-mouse Alexa 568 (1:500; Invitrogen, USA). After washing, Hoechst dye (Thermo Fisher Scientific, USA) was used to stain cells’ nuclei. The fluorescent signals were detected by BioTek Cytation 7 Cell Imaging Multi-Mode Reader (Agilent Technologies, USA).

### Serial Propagations of SARS-CoV-2

Vero E6, Vero E6/TMPRSS2, and Calu-3 cells were grown in six-well plates with seeding cell concentrations of 4 × 10^5^, 4 × 10^5^, and 6 × 10^5^ cells per well, respectively. The cells were cultured at 37 °C for 24 h when the cell density reached approximately 70–80% confluency.

Due to the limited availability of the clinical samples, TCID50 assay was used to quantify their viral titers on Vero E6/TMPRSS2 cells, and for the first passage of viral propagation (P1), 100 TCID_50_ of SARS-CoV-2-containing VTM was inoculated to the cells. After 1 h of viral absorption at 37 °C, fresh culture medium was added to the well (Vero E6: 2% FBS, 1× DMEM high glucose, and 100 μg/ml PS; Vero E6/TMPRSS2: 2% FBS, 1× DMEM low glucose, and 100 μg/ml PS; Calu-3: 10% FBS, 1× DMEM/F12, 1× Glutamax, and 100 μg/ml PS). Viral cultivation on Vero E6 cells, Vero E6/TMPRSS2 cells, and Calu-3 cells was carried out for 5 days, 2 days, and 5 days, respectively, when CPEs could be clearly observed (CPE grade 3+ or more). All images of viral CPEs were taken at 100× magnification using the Eclipse TS100 inverted microscope (Nikon, USA).

For subsequent passages (P2–P4), a total amount of 1,000 PFUs (evaluated by plaque assay on Vero cell monolayer, see below) of the virus-containing supernatant from the previous passage was inoculated into the cells. In all serial passages, the supernatants were harvested at day 5, day 2, and day 5 post-infection in Vero E6 cells, Vero E6/TMPRSS2 cells, and Calu-3 cells, respectively, at which point, again, clear CPEs similar to what observed in P1 could be seen. The collected supernatants were centrifuged at 4 °C, 3,000 rpm for 10 min and kept at −80 °C for virus genome sequencing and evaluation of the infectious virus titers. Each propagation experiment was performed in duplicate.

### Plaque Assay

The concentration of virus stock in each passage in the units of PFUs was determined as previously described (Kanjanasirirat et al. 2020). In brief, at 24 h prior to infection, Vero cells (ATCC®CCL-81™) were seeded in a six-well plate. Once the culture reached approximately 80% confluency, a serial dilution of the virus-containing supernatants was prepared for inoculation into a Vero cell monolayer. The cells were incubated for viral adsorption for 1 h at 37 °C incubator and then overlaid with 3 ml/well of MEM medium supplemented with 5% FBS and 1% agarose. The culture was incubated at 37 °C in 5% CO_2_ for 3 days. Thereafter, plaque phenotypes were visualized by staining with 0.33% Neutral Red solution (Sigma-Aldrich, USA) for 5 h. Plaque numbers were counted, and the virus titers were calculated as PFUs/ml.

### Focus Forming Assay

Infectious virus titers were measured using focus forming assay on Vero E6/TMPRSS2 cells (JCRB1819). Vero E6/TMPRSS2 cells were seeded in a 96-well at 2 × 10^4^ cells/well 24 h before infection. The cells were infected with a serial dilution of the virus-containing supernatants for 1 h at 37 °C. Then, 200 µl/well of overlay medium containing DMEM low glucose (Gibco, USA) supplemented with 2% FBS and 2% carboxymethylcellulose sodium salt (Sigma-Aldrich, USA) was added into the cells. The culture was incubated at 37 °C in 5% CO_2_ for 20 h. The cell monolayer was fixed with 4% formaldehyde for 1 h and was permeabilized in 0.5% Triton X-100 at room temperature for 10 min. After that, the cells were incubated with 1:2,500 rabbit monoclonal antibody against SARS-CoV-2 nucleoprotein (Sino Biological, China) at room temperature for 1 h, followed by three washes with PBST, and then staining with 1:1,000 horseradish peroxidase-conjugated goat anti-rabbit IgG secondary antibody (Agilent Technologies, USA) for 1 h. The cells were washed again with PBST three times. Subsequently, True-Blue peroxidase substrate (SeraCare Life science, USA) was inoculated into the cells and incubated for 10 min in a dark chamber, followed by a one-time wash with PBS. Foci numbers were observed under BioTek Cytation 7 Cell Imaging Multi-Mode Reader (Agilent Technologies, USA) and analyzed by Gen 5 version 3.11. The foci were counted and calculated as FFUs/ml.

### Whole Genome Sequencing

To sequence each passage stock, one sample of culture supernatant (200 µl) combined with cell lysate (50 µl) was harvested, and nucleic acid was extracted from the sample using the GenTi^TM32^ Automatic Extraction System (Advanced Viral DNA/RNA Extraction Kit, GeneAll, South Korea) according to the manufacturer's instructions. qRT-PCR of the spike gene was performed to quantify the cycle threshold (*C_t_*) values of the extracted RNA using a previously described PCR protocol (Fuk-Woo Chan et al. 2020). In brief, it was done using OneStep reverse transcription polymerase chain reaction (RT-PCR) Kit (Qiagen) in a Rotor-Gene Q Thermocycler (Qiagen). Forward (5ʹ-CCTACTAAATTAAATGATCTCTGCTTTACT-3ʹ) and reverse (5ʹ-CAAGCTATAACGCAGCCTGTA-3ʹ) primers targeting the spike gene were used. Each 20 μl reaction mixture contained 4 μl of 5× Qiagen OneStep RT-PCR buffer, 0.8 μl of deoxynucleoside triphosphate mix (10 mM each), 1.2 μl of each 10 μM forward and reverse primers, 0.8 μl of OneStep RT-PCR enzyme mix, 0.5 μl of SYBR Green (1:1,000), and 2 μl of extracted RNA and nuclease-free water. Reactions were incubated at 50 °C for 30 min, and 95 °C for 15 min, followed by 35 cycles at 94 °C for 30 s, 55 °C for 30 s, and 72 °C for 60 s, and then subjected to the melting curve analysis (65 °C for 1 min, followed by a gradual increase in temperature to 97 °C with continuous recording of fluorescence).

To sequence the samples, extracted RNA was diluted (if necessary), reverse-transcribed using SuperScript IV kit (Invitrogen Life Technologies, USA), followed by library preparation using the COVID-19 ARTIC v3 Illumina library construction protocol (dx.doi.org/10.17504/protocols.io.bibtkann). This protocol enriched SARS-CoV-2 genomic materials by many orders of magnitude by PCR amplification using 98 pairs of primers specific to SARS-CoV-2 (V.4 ARTIC V3 Panel, IDT, USA). The prepared libraries were then sequenced on a MiSeq (Illumina, USA; 250 paired-end).

### Intra-sample Genetic Variation Profiling

Sequencing read quality control was performed using fastp v 0.21.0 (Chen et al. 2018). Reads were first trimmed from the 3′ end to remove bases with average quality scores of <20 computed across a sliding window of eight nucleotides. Trimmed reads with fewer than 40 nucleotides were removed. Reads with average quality scores of <25 were also removed. This was done using the command *fastp –length_required 40 –average_qual 25 –cut_tail –cut_window_size 8 –cut_mean_quality 20*.

To compute site-wise sequencing depths and intra-sample genetic variation profiles, reads were mapped to the reference SARS-CoV-2 genome (RefSeq accession number: NC_045512.2) using bwa-mem2 v 2.2 (Vasimuddin et al. 2019) with a seed length setting of 29 and other parameter values under the default settings. Indel alignment quality scores were calculated and added to the alignment files using LoFreq v 2.1.5 (Wilm et al. 2012) with the *indelqual* command. Site-wise variant profiles were computed using the outputs from LoFreq's *plpsummary* command. Six variant types were called, including “A,” “T,” “C,” “G,” as well as “insertion” and “deletion.” Sites without read mapping, or spanning (in the case of deletion), were considered missing data.

### Detection of Potential Adaptive Changes

For each cell-line dataset, sites that had at least one virus sample displaying mutations at a frequency of >5% in any of the passage stocks in any of the propagation experiments were determined. For each of these sites, we fitted three mixed-effects logistic models to its mutation frequency data over time weighted by sequencing depths using the *relmatGlmer* function, implemented in the *lme4qtl* R package (Ziyatdinov et al. 2018; see supplementary note, Supplementary Material online). Datapoints supported by <30× sequencing depths were excluded from the model fittings. The first 30 bases of the 5′ UTR and the entire 3′ UTR were excluded from the analysis due to their low sequencing depths. Any variants that were not the major variant presenting in the original variant were collectively grouped together as the mutant variant in the model fittings.

In the simplest model (*M0*), we allowed the initial mutation frequencies (i.e., the model intercepts) to vary among the B.1.36.16 and AY.30 variants, while assuming that the mutant variant bares no selective advantage over the original variant. That is, the mutation selective advantage coefficients (i.e., the model slopes or the coefficients of the time variable, Δs) were assumed to be equal to zero for both of the variants. Potential sample-specific and experiment-specific random effects on the estimated intercepts were accounted for, while adjusting for the virus genetic similarity. The random effect of experimental replication was modeled to be nested within the random effect of the viral sample, in accordance with the experimental design.

In the other two alternative models, the initial mutation frequencies and the Δs values were estimated from the data and were allowed to vary among the B.1.36.16 and AY.30 variants. Likewise, potential sample-specific and experiment-specific random effects on the estimated intercepts were accounted for in both of these models, while adjusting for the virus genetic similarity. In one of the models, however, the Δs values were assumed to be shared and do not randomly vary significantly among viral samples of the same variant and experimental replicates (*M1*), while in the other model, the Δs values were allowed to vary randomly among viral samples and experimental replicates (*M2*).

At each site, the *anova* function in *R* was used to perform the likelihood ratio tests to identify the best-fit model. In the model comparisons, an analysis- and genome-wide Bonferroni multiple testing adjusted *P* value threshold of 5%/3 cell-line datasets/29,644 sites = 5.62 × 10^−5^% was used.

### 3D Protein Structure Modeling

Amino acid sequences of SARS-CoV-2's spike glycoprotein (NCBI Reference Sequence accession number: YP_009724390.1), nucleocapsid (YP_009724397.2), and NSP1 (YP_009725297.1) were submitted to AlphaFold2 at ColabFold (Jumper et al. 2021; Mirdita et al. 2022) for 3D protein structure modeling using the default parameter settings. The top model from each prediction was selected for drawing with the PyMOL Molecular Graphics System, Version 2.5.4 (Schrödinger and DeLano 2020). For the trimeric spike glycoprotein, the predicted structure of the domains surrounding the missing loops were superimposed to those of the cryogenic electron microscopy (cryo-EM) structure (Protein Data Bank (PDB) number: 6vsb; Hwang et al. 2020), and the predicted coordinates of the loops were added to the experimental structure without further optimization. The cryo-EM structure of the spike glycoprotein (PDB number: 6vsb; Hwang et al. 2020), and the membrane glycoprotein in a lipid nanodisc (PDB number: 8ctk; Dolan et al. 2022), as well as the crystal structure of NSP3 PLpro (PDB 7cjd; Gao et al. 2021), were obtained from the Protein Data Bank maintained by Research Collaboratory for Structural Bioinformatics (https://www.rcsb.org/; Berman et al. 2000).

## Supplementary Material

evad035_Supplementary_Data

## Data Availability

Raw sequencing data generated by this study are available from the Sequence Read Archive repository under the project accession number PRJNA853501 (clinical samples: SRR19880697–SRR19880700; cultured samples: SRR19880829–SRR19880912).

## Code Availability

R codes used to produce the results in this study are available from https://github.com/PAiewsakun/SARS-CoV-2-cell-culture-adaptation
