# Supplementary material for: Systematic Exploration of SARS-CoV-2 Adaptation to Vero E6, Vero E6/TMPRSS2, and Calu-3 Cells
Source: Genome Biol Evol. 2023 Feb 28;15(4):evad035. doi: 10.1093/gbe/evad035 (PMC10078795; doi:10.1093/gbe/evad035)
Supplement: evad035_Supplementary_Data [file evad035_supplementary_data.zip › Supplementary information legends.docx]

# Supplementary information

**Supplementary Data 1**. Infectious virus titres

**Supplementary Data 2**. Site wise variant profiles

**Supplementary Data 3**. Candidate sites with potential adaptation mutations

**Supplementary Data 4**. Potential adaptative indel changes around the spike protein’s multibasic cleavage site at the nucleotide level

**Supplementary Data 5**. Potential adaptative indel changes in the nucleocapsid protein’s flexible linker coding region at the nucleotide level

**Supplementary Data 6**. Potential adaptative indel changes in the NSP1 N-terminal domain coding region at the nucleotide level

**Supplementary Data 7**. Potential adaptative indel changes in the membrane glycoprotein’s third transmembrane domain coding region at the nucleotide level

**Supplementary Table 1**. Basic descriptions of the cell culture propagation experiments and sequencing summary statistics. The qRT-PCR cycle threshold (Ct) was determined by using a standard curve generated with a positive control containing SARS-CoV-2 at a concentration of 3×10^7^ virus copies/µl, determined by droplet digital PCR. Accession numbers of raw sequencing data are indicated.

**Supplementary Table 2.** Numbers of constant and polymorphic sites in the original and cultured samples. The counts excluded the first 30 bases of the 5’ UTR and the entire of 3’ UTR (229 sites). A site was considered a polymorphic site if it had two or more variants with frequencies of more than 1%. Polymorphic sites were further divided into two categories – bivariant (having only two variants) and multivariant (having more than two variants) sites.

**Supplementary Table 3.** Distributions of polymorphic sites by genetic change type. The distributions of polymorphic sites excluded the first 30 bases of the 5’ UTR and the entire of 3’ UTR (229 sites). A site was considered a polymorphic site if it had two or more variants with frequencies of more than 1 %. The direction of genetic change was inferred by assuming that the major variant detected in the original clinical sample was the original variant. For sites with more than one types of genetic changes, the counts are split between the multiple variant types, weighted by sequencing depths; hence, some of the counts are not integers.

**Supplementary Notes**. Modelling temporal dynamics of mutation frequency; Model fittings and comparisons; Computing the variance covariance matrix of the viral samples

**Supplementary Figure 1. Expression of ACE2 and TMPRSS2 in Vero E6, Vero E6/TMPRSS2, and Calu-3 cells. (a)** Immunofluorescence staining of ACE2 and TMPRSS2 in Vero E6, Vero E6/TMPRSS2, and Calu-3 cells was performed by using a rabbit polyclonal antibody recognizing ACE2 with goat anti-rabbit Alexa 488 (Green) and a mouse monoclonal antibody specific to TMPRSS2 with goat anti-mouse Alexa 568 (Red). Nuclear DNA was stained with Hoechst dye (Blue). The fluorescent signals were detected by the BioTek Cytation 7 Cell Imaging Multimode Reader (Agilent Technologies, USA). The data are representative examples of three independent experiments. Scale bars indicate 200 μm. Zoomed-in images of the randomly selected box sections are shown in the most-right column. Quantification of ACE2 **(b)** and TMPRSS2 **(c)** levels based on the percentage of fluorescence-positive cells and the mean fluorescence intensity/MFI was analysed in each cell type used in this study. Multiple comparison was performed using a one-way analysis of variance (ANOVA) with Tukey post-hoc analysis. *P*-value *< 0.001* (***) was considered statistical significance (GraphPad Prism 9).

**Supplementary Figure 2. Cytopathic effects observed in passage 1.** Panels **a**, **f** and **k** are uninfected Vero E6 cells, Vero E6/TMPRSS2 cells, and Calu-3 cells, respectively. In viral propagation experiment, Vero E6 cells **(b–e)**, Vero E6/TMPRSS2 cells **(g–j)** and Calu-3 cells **(l–o)** were infected with 1000 plaque-forming units of the two B.1.36.16 samples (73NLt and CV130) and the two Delta AY.30 samples (NH783 and OTV54) for 5 days, 2 days, and 5 days, respectively. All images were taken at 100× magnification using the Eclipse TS100 inverted microscope (Nikon, USA).

**Supplementary Figure 3. Temporal dynamics of potential adaptive changes detected in viruses propagated in Vero E6 cells**. Plot strip labels indicate the position at which the signals were detected together with its associated gene and coding domain information. Samples with estimated ∆s values greater than 0, and mutation frequencies greater than 5% in any passage stocks are shown with solid colours, otherwise transparent. Estimated $\Delta s$ values for the two virus variants together with associated nucleotide and amino acid mutational changes detected, as well as the experiments showing strong signals at each site can be found in **Supplementary Data 3**.

**Supplementary Figure 4. Temporal dynamics of potential adaptive changes detected in viruses propagated in Vero E6/TMPRSS2 cells.** See legend to **Supplementary Figure 3.**

**Supplementary Figure 5. Temporal dynamics of potential adaptive changes detected in viruses propagated in Calu-3 cells.** See legend to **Supplementary Figure 3**.
