# Supplementary material for: Systematic Exploration of SARS-CoV-2 Adaptation to Vero E6, Vero E6/TMPRSS2, and Calu-3 Cells
Source: Genome Biol Evol. 2023 Feb 28;15(4):evad035. doi: 10.1093/gbe/evad035 (PMC10078795; doi:10.1093/gbe/evad035)
Supplement: evad035_Supplementary_Data [file evad035_supplementary_data.zip › Supplementary Notes and Figures.pdf]

Supplementary Figures and Notes

Systematic exploration of SARS-CoV-2 adaptation to Vero E6, Vero E6/TMPRSS2, and Calu-3 cells

Authors

Pakorn Aiewsakun[1,2]*, Worakorn Phumiphanjarphak[1,2], Natali Ludowyke[1], Priyo Budi Purwono[1], Suwimon Manopwisedjaroen[1], Chanya Srisaowakarn[1], Supanuch Ekronarongchai[1], Ampa Suksatu[1], Jirundon Yuvaniyama[3], Arunee Thitithanyanont[1,2]*

Author Affiliations

1. Department of Microbiology, Faculty of Science, Mahidol University, 272, Rama VI Road, Ratchathewi, Bangkok, 10400, Thailand.
2. Pornchai Matangkasombut Center for Microbial Genomics, Department of Microbiology, Faculty of Science, Mahidol University, 272, Rama VI Road, Ratchathewi, Bangkok, 10400, Thailand.
3. Department of Biochemistry and Center for Excellence in Protein and Enzyme Technology, Faculty of Science, Mahidol University, 272, Rama VI Road, Ratchathewi, Bangkok, 10400, Thailand.
* corresponding authors

Correspondence

pakorn.aie@mahidol.ac.th; arunee.thi@mahidol.ac.th

Supplementary Figure 1

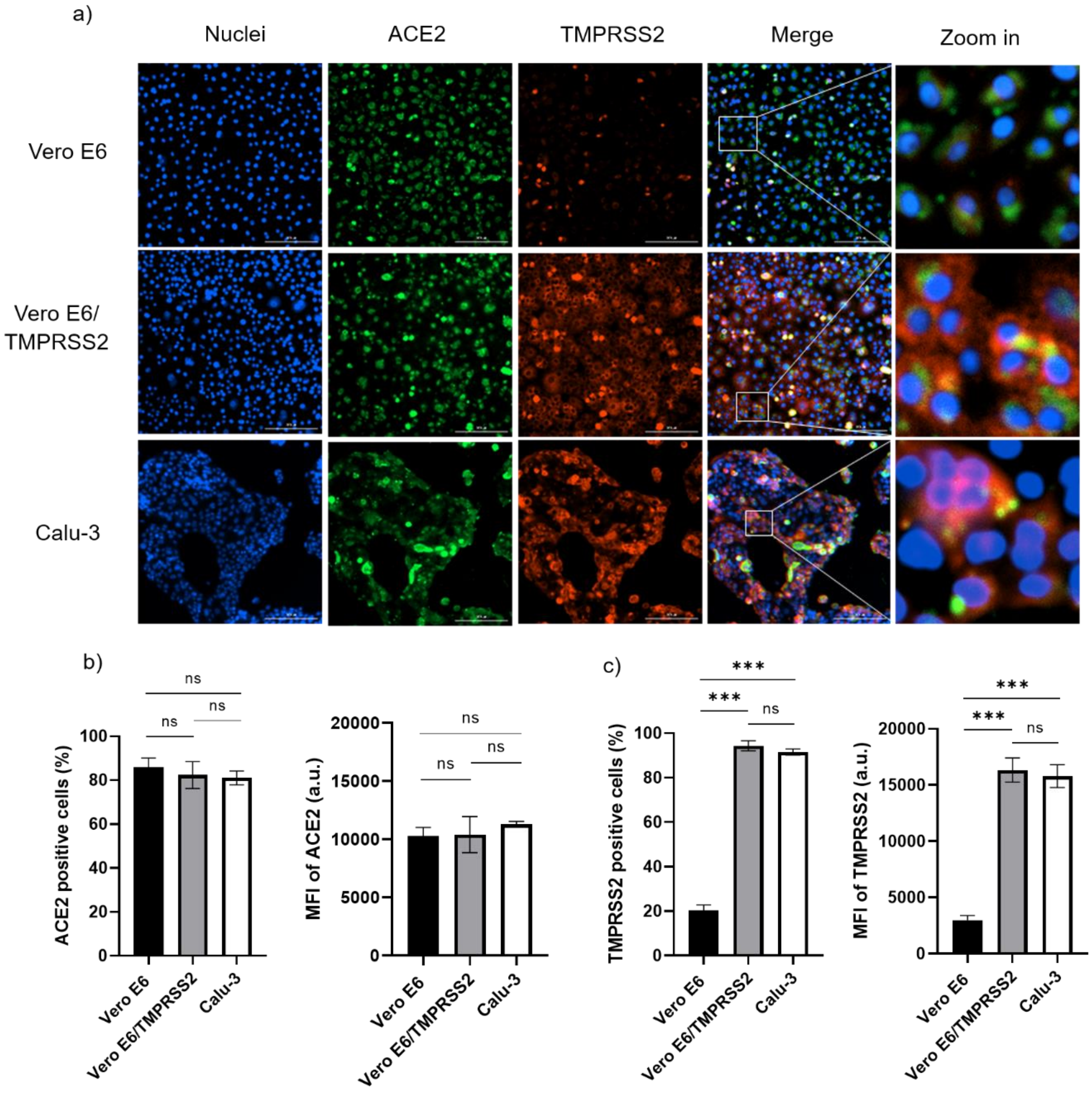

Supplementary Figure 1. Expression of ACE2 and TMPRSS2 in Vero E6, Vero E6/TMPRSS2, and Calu-3 cells. **(a)** Immunofluorescence staining of ACE2 and TMPRSS2 in Vero E6, Vero E6/TMPRSS2, and Calu-3 cells was performed by using a rabbit polyclonal antibody recognizing ACE2 with goat anti-rabbit Alexa 488 (Green) and a mouse monoclonal antibody specific to TMPRSS2 with goat anti-mouse Alexa 568 (Red). Nuclear DNA was stained with Hoechst dye (Blue). The fluorescent signals were detected by the BioTek Cytation 7 Cell Imaging Multimode Reader (Agilent Technologies, USA). The data are representative examples of three independent experiments. Zoomed-in images of the randomly selected box sections are shown in the most-right column. Scale bars indicate 200 µm. Quantification of ACE2 **(b)** and TMPRSS2 **(c)** levels based on the percentage of fluorescence-positive cells and the mean fluorescence intensity/MFI was analysed in each cell type used in this study. Multiple comparison was performed using a one-way analysis of variance (ANOVA) with Tukey post-hoc analysis. P-value < *0.001* (***) was considered statistical significance (GraphPad Prism 9).

Supplementary Figure 2

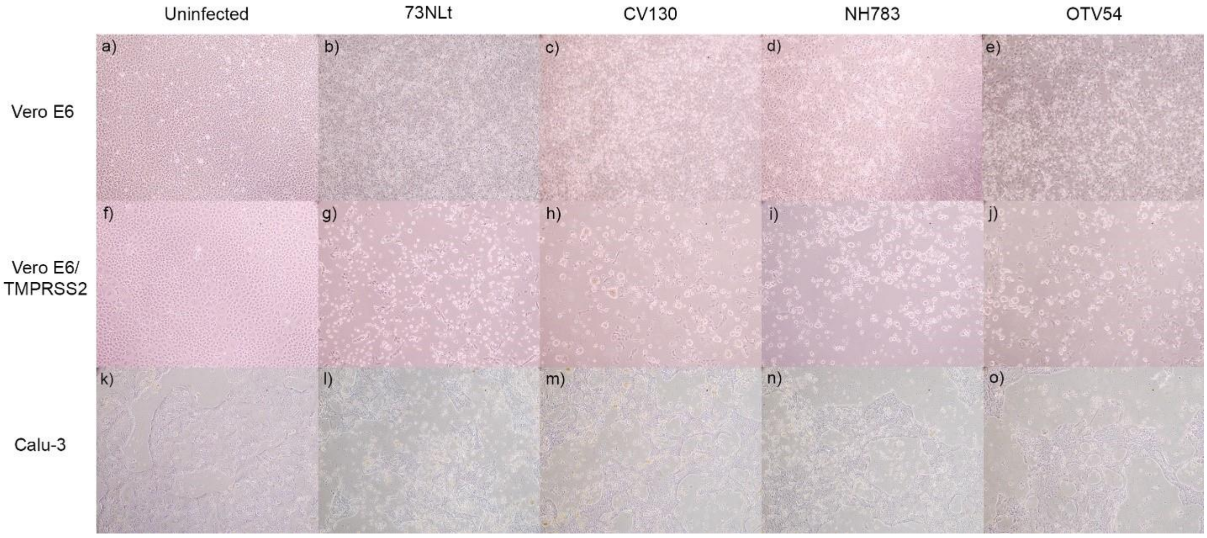

Supplementary Figure 2. Cytopathic effects observed in passage 1. Panels **a**, **f** and **k** are uninfected Vero E6 cells, Vero E6/TMPRSS2 cells, and Calu-3 cells, respectively. In viral propagation experiment, Vero E6 cells **(b–e)**, Vero E6/TMPRSS2 cells **(g–j)** and Calu-3 cells **(l–o)** were infected with 1000 plaque-forming units of the two B.1.36.16 samples (73NLt and CV130) and the two Delta AY.30 samples (NH783 and OTV54) for 5 days, 2 days, and 5 days, respectively. All images were taken at 100× magnification using the Eclipse TS100 inverted microscope (Nikon, USA).

Supplementary Figure 3

[See next page]

Supplementary Figure 3. Temporal dynamics of potential adaptive changes detected in viruses propagated in Vero E6 cells. Plot strip labels indicate the position at which the signals were detected together with its associated gene and coding domain information. Samples with estimated Δs values greater than 0, and mutation frequencies greater than 5% in any passage stocks are shown with solid colours, otherwise transparent. Estimated Δs values for the two virus variants together with associated nucleotide and amino acid mutational changes detected, as well as the experiments showing strong signals at each site can be found in **Supplementary Data 3**.

Temporal dynamics of potential Vero E6 adaptive mutations

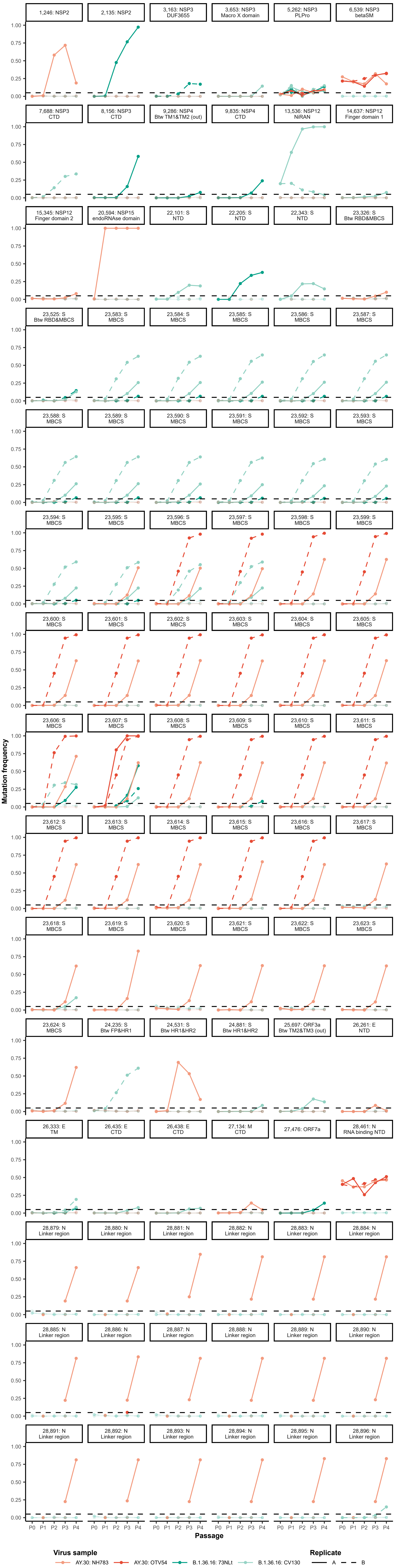

Supplementary Figure 4

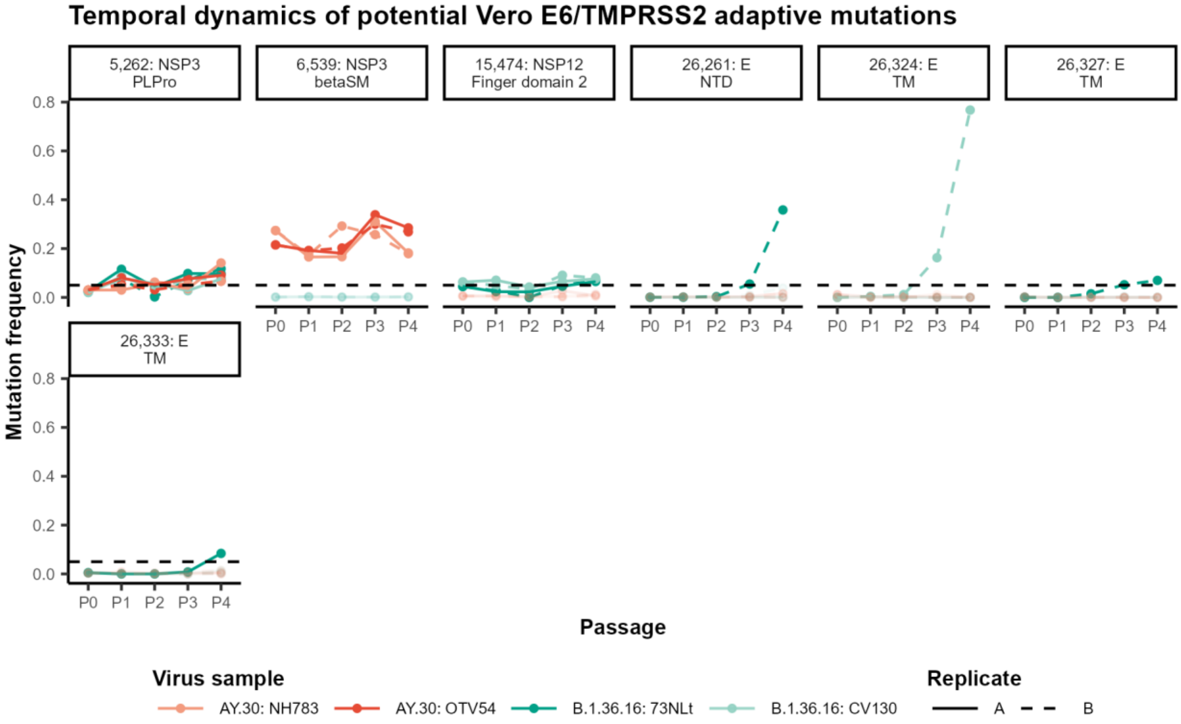

Supplementary Figure 4. Temporal dynamics of potential adaptive changes detected in viruses propagated in Vero E6/TMPRSS2 cells. See legend to **Supplementary Figure 3.**

Supplementary Figure 5

[See next page]

Supplementary Figure 5. Temporal dynamics of potential adaptive changes detected in viruses propagated in Calu-3 cells. See legend to **Supplementary Figure 3.**

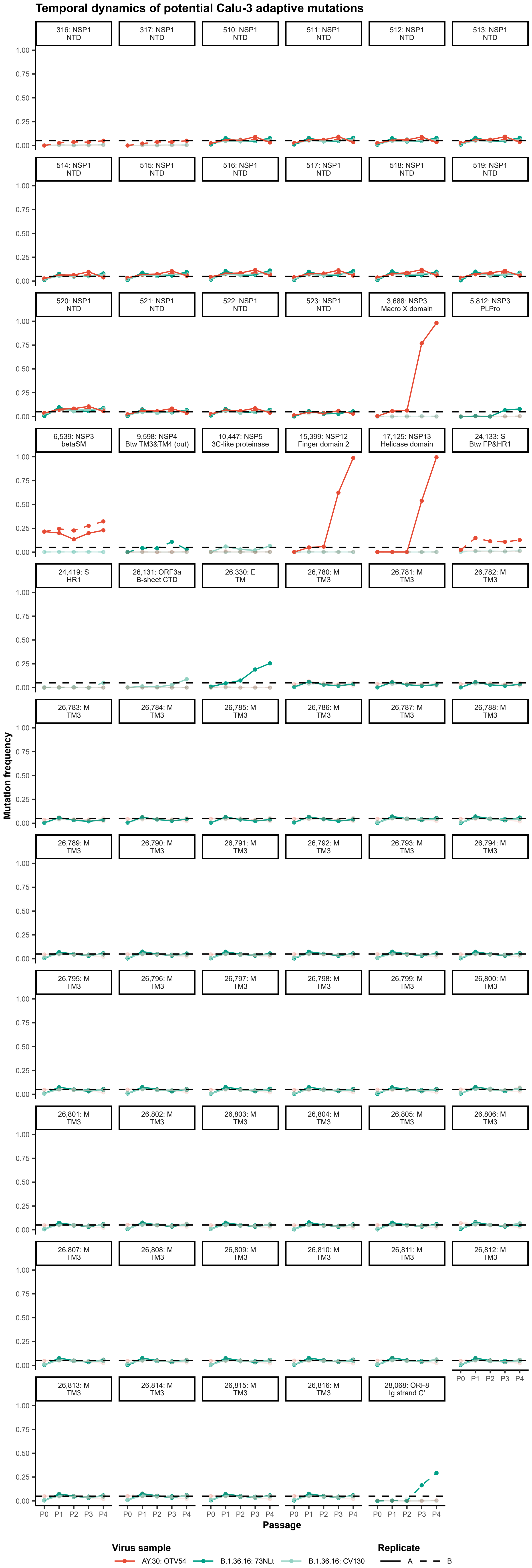
Temporal dynamics of potential Calu-3 adaptive mutations
316: NSP1 NTD
317: NSP1 NTD
510: NSP1 NTD
511: NSP1 NTD
512: NSP1 NTD
513: NSP1 NTD
514: NSP1 NTD
515: NSP1 NTD
516: NSP1 NTD
517: NSP1 NTD
518: NSP1 NTD
519: NSP1 NTD
520: NSP1 NTD
521: NSP1 NTD
522: NSP1 NTD
523: NSP1 NTD
3,688: NSP3 Macro X domain
5,812: NSP3 PLPro
6,539: NSP3 betaSM
9,598: NSP4 Btw TM3&TM4 (out)
10,447: NSP5 3C-like proteinase
15,399: NSP12 Finger domain 2
17,125: NSP13 Helicase domain
24,133: S Btw FP&HR1
24,419: S HR1
26,131: ORF3a B-sheet CTD
26,330: E TM
26,780: M TM3
26,781: M TM3
26,782: M TM3
26,783: M TM3
26,784: M TM3
26,785: M TM3
26,786: M TM3
26,787: M TM3
26,788: M TM3
26,789: M TM3
26,790: M TM3
26,791: M TM3
26,792: M TM3
26,793: M TM3
26,794: M TM3
26,795: M TM3
26,796: M TM3
26,797: M TM3
26,798: M TM3
26,799: M TM3
26,800: M TM3
26,801: M TM3
26,802: M TM3
26,803: M TM3
26,804: M TM3
26,805: M TM3
26,806: M TM3
26,807: M TM3
26,808: M TM3
26,809: M TM3
26,810: M TM3
26,811: M TM3
26,812: M TM3
26,813: M TM3
26,814: M TM3
26,815: M TM3
26,816: M TM3
28,068: ORF8 Ig strand C'
Mutation frequency
0.00
0.25
0.50
0.75
1.00
Passage
P0 P1 P2 P3 P4
Virus sample
AY.30: OTV54
B.1.36.16: 73NLt
B.1.36.16: CV130
Replicate
A
B

Modelling temporal dynamics of mutation frequency

In this study, we assumed that all co-existing virus variants in a cell culture could grow exponentially and indefinitely without any resource limitation, of which their overall population growth rates could be modelled as follows:

$$\frac{dN_i}{dt} = s_i N_i \qquad \textbf{Eq.1}$$

where N_i denotes the population size of variant i; t is time; and s_i is the intrinsic growth rate of variant i. Under this model, the time evolution of the population size of variant i, N_i^t, could be described mathematically as follows:

$$N_i^t = N_i^0 e^{s_i t} \qquad \textbf{Eq.2}$$

where N_i^0 is the initial population size of variant i at time $t = 0$. Given the dataset that we had, we deemed it reasonable to model each site as having just two variants, namely the original variant, ori – the major variant presenting in the original clinical sample, and the mutant variant, mut – any other variants that were not ori variant grouped together (see **Main text**). Based on this, at each site, we could model the time evolution of the population size of the two variants as follows:

$$N_{mut}^t = N_{mut}^0 e^{s_{mut} t} \qquad \textbf{Eq.3}$$
$$N_{ori}^t = N_{ori}^0 e^{s_{ori} t} \qquad \textbf{Eq.4}$$

Dividing **Eq.3** with **Eq.4** to obtain that;

$$\frac{N_{mut}^t}{N_{ori}^t} = \frac{N_{mut}^0 e^{s_{mut} t}}{N_{ori}^0 e^{s_{ori} t}};$$
$$\frac{N_{mut}^t / N^t}{N_{ori}^t / N^t} = \frac{(N_{mut}^0 / N^0) e^{s_{mut} t}}{(N_{ori}^0 / N^0) e^{s_{ori} t}};$$
$$\frac{f_{mut}^t}{f_{ori}^t} = \frac{f_{mut}^0 e^{s_{mut} t}}{f_{ori}^0 e^{s_{ori} t}} \qquad \textbf{Eq.5}$$

where N^t is the total population size at time t; and f_i^t is the frequency of variant i at time t. Notice that under the assumption that there were only two variants in the population, $f_{ori}^t + f_{mut}^t = 1$; thus, we could rewrite and rearrange **Eq.5** as follows:

$$\frac{f_{mut}^t}{1 - f_{mut}^t} = \frac{f_{mut}^0 e^{s_{mut} t}}{(1 - f_{mut}^0) e^{s_{ori} t}};$$
$$\ln \frac{f_{mut}^t}{1 - f_{mut}^t} = \ln \frac{f_{mut}^0}{1 - f_{mut}^0} + t\Delta s;$$
$$\text{logit}(f_{mut}^t) = \text{logit}(f_{mut}^0) + t\Delta s;$$
$$f_{mut}^t = \text{logit}^{-1}(\text{logit}(f_{mut}^0) + t\Delta s) \qquad \textbf{Eq.6}$$

where $\text{logit}(.)$ is a logit function (i.e. the inverse of the logistic function), and Δs is the difference between the intrinsic growth rates of the two variants; $\Delta s = s_{mut} - s_{ori}$. In this work, we used **Eq.6** as the basis of culture adaption mutation detection – a site with cell culture adaptation mutations should show a significantly positive Δs value. This term could also be seen as a mutation selective advantage coefficient.

Eq.6 is a simple logistic function. Thus, to estimate Δs values for mutations occurring at a particular site in the two virus variants, a generalised linear mixed model with a logit link function, also known as a mixed-effects logistic model, can be fitted to their mutation frequency data over passages, weighted by sequencing depths. Mapping to **Eq.6**, the intercept of the model linear predictors is the $\text{logit}(f_{mut}^{0})$ term, and the coefficient of the passage variable, i.e. the model slope, is the Δs term. In this work, we asserted that the data obtained from original clinical samples were collected at $t = 0$, and those obtained from passage stocks 1 (P1) to 4 (P4) were collected at $t = 1, \ldots, 4$, respectively. In other words, we assumed that the data collected at different time points were temporally equally spaced. We deemed this reasonable as, in each propagation experiment, all viral samples had the same period of times to grow in the culture in each passage. In the model fittings, we also allowed the intercept and / or the slope of the model to vary randomly among the four viral samples, and the experimental replicates, while adjusting for the virus phylogenetic relatedness. The random effect of experimental replication was nested within the random effect of the virus sample.

Model fittings and comparisons

For each cell line dataset, sites that had at least one viral sample displaying mutations at a collective frequency of > 5% in any of the passage stocks in any of the propagation experiments were determined. For each of these sites, we fitted three mixed-effects logistic models to its mutation frequency data over time weighted by sequencing depths using the *relmatGlmer* function, implemented in the *lme4qtl* R package (Ziyatdinov et al. 2018). Datapoints supported by < 30× sequencing depths were excluded from the model fittings. The first 30 bases of the 5' UTR and the entire 3' UTR were excluded from the analysis due to their low sequencing depths. Any variants that were not the major variant presenting in the original variant were collectively grouped together as the mutant variant in the model fittings.

In the simplest model (**M0**), we allowed the initial mutation frequencies (i.e. the model intercepts) to vary among the B.1.36.16 and AY.30 variants, while assuming that the mutant variant bares no selective advantage over the original variant. That is, the mutation selective advantage coefficients (i.e. the model slopes, or the coefficients of the time variable, Δs) were assumed to be equal to zero for both of the virus variants. Potential sample-specific and experiment-specific random effects on the estimated intercepts were accounted for, while adjusting for the phylogenetic structure of the four viral samples. The random effect of experimental replication was modelled to be nested within the random effect of the viral sample, and the virus phylogenetic structure was represented by the variance covariance matrix of the viral sample phylogeny normalised to have a determinant of 1 (see **Computing the variance covariance matrix of the viral samples**). The R code is shown below:

```
M0 <- relmatGlmer(
formula = fMut ~ variant - 1 + (1|sample/replicate),
weight = depth,
data = subset_variant_table_dat,
relmat = list(sample = VPhy),
family = binomial
)
```

where `fMut` is the mutation frequency variable; `variant` is the virus variant factor variable; `sample` is the viral sample factor variable; `replicate` is the experimental replicate factor variable; `depth` is the sequencing depth; `subset_variant_table_dat` is the data frame that holds all of these data of the site under the analysis; and `VPhy` is the normalised variance covariance matrix of the viral sample phylogeny. The term "`-1`" was added to the formula to force the program to compute separate intercepts for each of the virus variants.

In the other two alternative models, the initial mutation frequencies and the Δs values were estimated from the data, and were allowed to vary among the B.1.36.16 and AY.30 variants. Likewise, potential sample-specific and experiment-specific random effects on the estimated intercepts were accounted for in both of these models, while adjusting for the virus genetic similarity. In one of the models, the Δs values were assumed to be shared among viral samples of the same variant and experimental replicates (**M1**), while in the other model, the Δs values were allowed to vary randomly among viral samples and experimental replicates (**M2**). Below is the R code to fit the two models.

```
M1 <- relmatGlmer(
formula = fMut ~ variant - 1 + variant:t + (1|sample/replicate),
weights = depth,
data = subset_variant_table_dat,
relmat = list(sample = Vphy),
family = binomial
)

M2 <- relmatGlmer(
```

```
formula = fMut ~ variant - 1 + variant:t + (1+t|sample/replicate),
data = subset_variant_table_dat,
weights = depth,
relmat = list(sample = Vphy),
family = binomial
)
```

where `t` is the time variable. In the model fittings, we asserted that the data obtained from original clinical samples were those collected at t = 0, and those obtained from passage stocks 1 to 4 were collected at t = 1,…,4, respectively.

At each site, the *anova* function in *R* was used to perform sequential likelihood ratio tests to identify the best-fit model. **M0** was first compared against **M1**, and if the mutation frequency dynamics was found to be significantly better described by **M1**, **M1** was then compared against **M2**. Sites that had genetic variations with a significant selective advantage for at least one of the virus variants, i.e. best described by either **M1** or **M2**, were determined and reported (**Supplementary Data 3**). In the model comparisons, an analysis- and genome-wide Bonferroni multiple-testing adjusted p value threshold of 5% / 3 cell line datasets / 29,644 sites = 5.62×10^{-7} % was used.

Computing the variance covariance matrix of the viral samples

The phylogeny of the four viral samples was reconstructed in order to compute the variance covariance matrix of the viruses. We, first, constructed consensus sequences of the four viral samples obtained from the original clinical samples from their read mapping alignments against the SARS-CoV-2 Wuhan-Hu-1 reference genome (RefSeq accession number: NC_045512.2). Variants with respect to the reference genome were called by using the *call* command implemented in LoFreq v 2.1.5 (Wilm et al. 2012) with the default settings and the *no-default-filter* option. Variants with more than 50% frequencies were then substituted in the place of reference alleles to create our viral sample consensus sequences using the *consensus* command in BCFtools v 1.12-57-g0c2765b (Danecek and McCarthy 2017). For each sample, the process was iterated by using the newly constructed sequence as the genome mapping reference until no alternative variants with more than 50% frequencies were found.

Together with the reference Wuhan-Hu-1 SARS-CoV-2 genome, a multiple sequence alignment of the virus whole genome sequences was made, and was used to reconstruct a maximum likelihood tree. The maximum likelihood tree reconstruction was performed by using IQ-TREE (Minh et al. 2020). TPM2u+F model was determined to be the best-fit nucleotide substitution model under the Bayesian information criterion for the alignment data by ModelFinder (Kalyaanamoorthy et al. 2017), and was used for the tree reconstruction. The bootstrap clade support was computed based on 1,000 pseudoreplicate datasets with ultrafast bootstrap approximation. The tree was rooted on the branch leading to the Wuhan-Hu-1 genome, one of the earliest SARS-CoV-2 genomes to be sequenced in the pandemic, and the sequence was then removed from tree. The variance covariance matrix of the viruses was computed using the *vcv* function in the *ape* R package and was normalised to have a determinant of 1.

References

Danecek P, McCarthy SA. 2017. BCFtools/csq: haplotype-aware variant consequences. *Bioinformatics* 33:2037–2039.

Kalyaanamoorthy S, Minh BQ, Wong TKF, Von Haeseler A, Jermiin LS. 2017. ModelFinder: Fast model selection for accurate phylogenetic estimates. *Nat. Methods* 14:587–589.

Minh BQ, Schmidt HA, Chernomor O, Schrempf D, Woodhams MD, von Haeseler A, Lanfear R. 2020. IQ-TREE 2: New models and efficient methods for phylogenetic inference in the genomic era. *Mol. Biol. Evol.* 37:1530–1534.

Wilm A, Aw PPK, Bertrand D, Yeo GHT, Ong SH, Wong CH, Khor CC, Petric R, Hibberd ML, Nagarajan N. 2012. LoFreq: A sequence-quality aware, ultra-sensitive variant caller for uncovering cell-population heterogeneity from high-throughput sequencing datasets. *Nucleic Acids Res.* 40:11189–11201.

Ziyatdinov A, Vázquez-Santiago M, Brunel H, Martinez-Perez A, Aschard H, Soria JM. 2018. lme4qtl: Linear mixed models with flexible covariance structure for genetic studies of related individuals. *BMC Bioinformatics* 19:68.
